# Vasopressin in Hemorrhagic Shock: A Systematic Review and Meta-Analysis of Randomized Animal Trials

**DOI:** 10.1155/2014/421291

**Published:** 2014-09-01

**Authors:** Andrea Pasquale Cossu, Paolo Mura, Lorenzo Matteo De Giudici, Daniela Puddu, Laura Pasin, Maurizio Evangelista, Theodoros Xanthos, Mario Musu, Gabriele Finco

**Affiliations:** ^1^Department of Medical Sciences “M. Aresu”, University of Cagliari, SS.554 Bivio per Sestu, 09042 Monserrato, Italy; ^2^Department of Anesthesia and Intensive Care, Vita-Salute San Raffaele University, Via Olgettina 60, 20132 Milano, Italy; ^3^Department of Anesthesia and Intensive Care, Catholic University, Via Giuseppe Moscati 31, 00198 Roma, Italy; ^4^MSc Program “Cardiopulmonary Resuscitation”, University of Athens, Medical School, Hellenic Society of Cardiopulmonary Resuscitation, 75 Mikras Asias Street, 11527 Athens, Greece

## Abstract

*Objective.* The latest European guidelines for the management of hemorrhagic shock suggest the use of vasopressors (norepinephrine) in order to restore an adequate mean arterial pressure when fluid resuscitation therapy fails to restore blood pressure. The administration of arginine vasopressin (AVP), or its analogue terlipressin, has been proposed as an alternative treatment in the early stages of hypovolemic shock.* Design.* A meta-analysis of randomized controlled animal trials.* Participants.* A total of 433 animals from 15 studies were included.* Interventions.* The ability of AVP and terlipressin to reduce mortality when compared with fluid resuscitation therapy, other vasopressors (norepinephrine or epinephrine), or placebo was investigated.* Measurements and Main Results.* Pooled estimates showed that AVP and terlipressin consistently and significantly improve survival in hemorrhagic shock (mortality: 26/174 (15%) in the AVP group versus 164/259 (63%) in the control arms; OR = 0.09; 95% CI 0.05 to 0.15; *P* for effect < 0.001; *P* for heterogeneity = 0.30; *I*_2_ = 14%).* Conclusions.* Results suggest that AVP and terlipressin improve survival in the early phases of animal models of hemorrhagic shock. Vasopressin seems to be more effective than all other treatments, including other vasopressor drugs. These results need to be confirmed by human clinical trials.

## 1. Introduction

Trauma is the principal cause of death for people under 35 years of age, with more than 5 million injury-related deaths every year in the world. Approximately 30% of these deaths can be attributed to hemorrhagic shock [1, 2]. Untreated prehospital hemorrhagic shock is one of the leading causes of cardiac arrest [3, 4]. Appropriate management and treatment are necessary to prevent adverse events and outcomes [5–7]. The early phase of hemorrhagic shock is characterized by a vasoconstrictive response and if the shock is left untreated it can lead to vasodilation that does not respond to conventional resuscitation strategies [8, 9]. Prehospital hemorrhagic shock treatment should be focused on maintaining adequate mean arterial pressure (MAP) along with organ perfusion up until arrival at the hospital [10, 11].

Small volume resuscitation with colloids or hyperoncotic fluids may be useful during early phases of uncontrolled bleeding [12–14]. Recent international guidelines suggest that vasopressors may also be required to maintain tissue perfusion where fluid resuscitation itself does not achieve the expected goal [15].

Arginine vasopressin (AVP) is an endogenous neurohypophysial hormone with an antidiuretic function. The most important AVP release stimulus is the plasma osmolality variation followed by blood pressure variations [16–18]. AVP also suppresses nitric oxide (NO) production [19]. The AVP release may also be suppressed by increased levels of norepinephrine and the increased release of NO from vascular endothelium of the posterior pituitary gland [20, 21]. Terlipressin is a long-acting synthetic analogue of AVP, proposed in the septic shock management as a rescue therapy, when adequate MAP values are difficult to reach with standard therapy. It is characterized by a longer duration of action and a higher selectivity on the V_1_ receptors that limits the edemigenous effect mediated by the V_2_ receptors differently from what its native counterpart does [22]. AVP and terlipressin can be both used with the aim of reaching the desired MAP target or reducing the norepinephrine dosage [23, 24].

In animal models in which severe uncontrolled blood loss has been induced, the administration of AVP has shown improvement in survival, neurologic outcome, and enhanced hemodynamic performance [25–27]. During the irreversible phase of hemorrhagic shock, unresponsive to fluids and catecholamines administration, AVP can mediate peripheral vasoconstriction through V_1_ receptors [13, 28, 29]. AVP works primarily on arterioles in extracerebral tissues, with less constriction action on coronary and renal vessels with potential vasodilatory effect on cerebral and pulmonary flow [30]. Recent animal studies have shown that AVP treatment can achieve hemodynamic optimization during prehospital hemorrhagic shock, while fluids and catecholamines showed neither improvement of hemodynamic parameters nor survival [1, 31, 32].

AVP use is associated with some adverse effects such as ischemic complications especially in cardiac, splanchnic, and skin circulation [33]. The decreased gut perfusion may determine tissue necrosis with subsequent translocation of bacteria that promotes the development of sepsis in the postresuscitation phase [34]. The increased expression of the V_1_ receptor subtype in trauma brain injury might promote the development of cerebral edema [8, 35].

To evaluate the impact on survival of V_1_ receptor agonists in hypovolemic refractory shock, we conducted a systematic review and meta-analysis of data pooled from existing trials comparing AVP or terlipressin and conventional shock management in mammals.

## 2. Materials and Methods

### 2.1. Search Strategy

All randomized animal trials using AVP or terlipressin in hypovolemic shock were identified. Relevant studies were independently searched by two trained investigators in Google Scholar and PubMed (updated November 4, 2013). The full PubMed search strategy, including keywords AVP, arginine vasopressin, terlipressin, and hemorrhagic and hypovolemic shock, was developed according to Biondi-Zoccai et al. and is available in the Appendix [36].

### 2.2. Study Selection

References obtained from databases and literature searches were first examined independently at the title/abstract level by two investigators, with divergences resolved by consensus and then, if potentially pertinent, retrieved as a complete article.

Inclusion criteria for potentially relevant studies were random allocation to treatment; animal experimental design; comparison of AVP or terlipressin (with or without fluid administration) versus placebo or fluids or catecholamines or both fluids plus catecholamines. Exclusion criteria were duplicate publications, human trials, and studies with no data on survival. Two investigators selected studies for the final analysis by independently assessing compliance to the selection criteria. Divergences from the selection criteria were resolved by consensus.

### 2.3. Data Abstraction and Study Characteristics

Two investigators independently extracted data on the study design, experimental setting, dosages of AVP or terlipressin, and experimental duration, with divergences resolved by consensus. If the required data could not be retrieved from the published report, at least two separate attempts to contact the original authors were made.

The primary end-point was mortality at the longest available follow-up. In addition, we performed further subanalysis comparing animals treated with AVP (or terlipressin) with those treated, respectively, with placebo, fluid resuscitation, and other vasoconstrictive drugs.

### 2.4. Data Analysis and Synthesis

Computations were performed with RevMan 4.2 [35]. Binary outcomes from individual studies were analyzed to compute individual odds ratios (ORs) with pertinent 95% confidence intervals (CIs), and a pooled summary effect estimate was calculated by means of the Mantel-Haenszel method and the fixed effect model in case of low statistical inconsistency (*I* ^2^ < 25%) or the random-effect model in case of moderate or high statistical inconsistency (*I* ^2^ > 25%) [37]. Statistical heterogeneity and inconsistency were measured using Cochran *Q* tests and *I* ^2^ (by Higgins and Thompson), respectively [38]. Statistical significance was set at 2-tailed 0.05 for hypothesis testing and at 0.10 for heterogeneity testing. According to Higgins et al., the *I* ^2^ values around 25%, 50%, and 75% were considered to represent, respectively, low, moderate, and severe statistical inconsistency [38].

The risk of publication bias was assessed by visual inspection of the funnel plot for mortality. Sensitivity analyses were performed by sequentially removing each study and reanalysing the remaining dataset (producing a new analysis for each study removed) and by analysing only data from studies with low risk of bias.

## 3. Results

### 3.1. Study Characteristics

**Figure 1:**
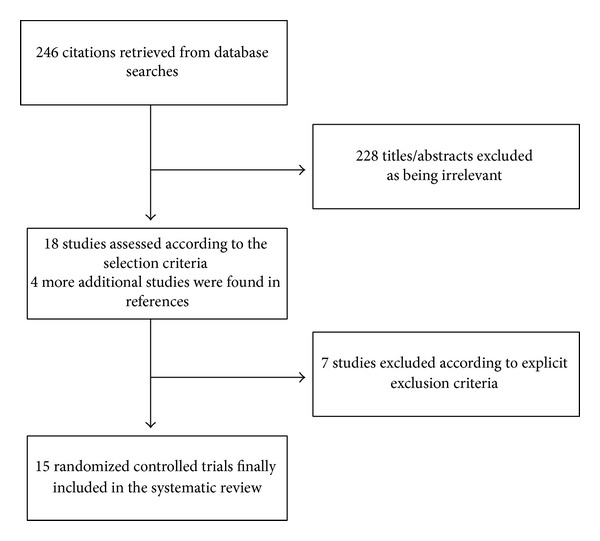
Flow diagram of the systematic review process.

Database searches, backwards snowballing, and contacts with experts yielded a total of 246 citations. After excluding nonpertinent titles or abstracts, 22 studies were retrieved in complete form and assessed according to the selection criteria (Figure 1). Seven studies were further excluded for the absence of survival data. Fifteen eligible trials were included in the final analysis.

The 15 included studies randomized 433 animals, 174 to AVP (14 trials) or terlipressin (one trial) and 259 to control (placebo, vasopressors, or fluid resuscitation). The included trials were conducted on pigs (12 studies) and on rats (three studies). All manuscripts were published in indexed journals. Detailed study characteristics are summarized in Table 1.

### 3.2. Quantitative Data Synthesis

**Figure 2:**
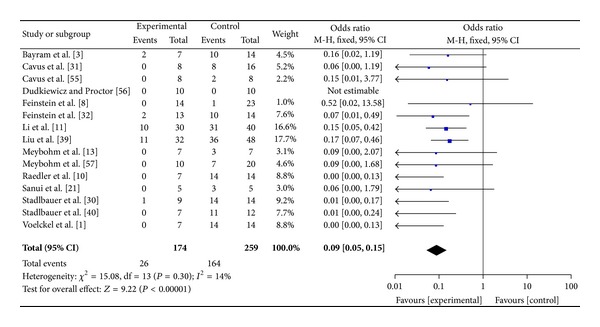
AVP or terlipressin versus all other strategies (fluid resuscitation, vasoconstrictors, and placebo).

The overall analysis showed that AVP/terlipressin were associated with a reduction in animal mortality (26/174 (15%) in the AVP/terlipressin group versus 164/259 (63%) in the control arms; OR = 0.09 (95% CI 0.05–0.15); *P* for effect < 0.001; *P* for heterogeneity = 0.30; *I* ^2^ = 14%) (Figure 2). When studies were grouped to either fluid resuscitation, placebo, norepinephrine, or other vasoconstrictive drugs as a comparator, administration of AVP/terlipressin was still associated with a reduction in mortality. (see Supplementary Figures 6(b)–6(e) available online at http://dx.doi.org/10.1155/2014/421291).

**Figure 3:**
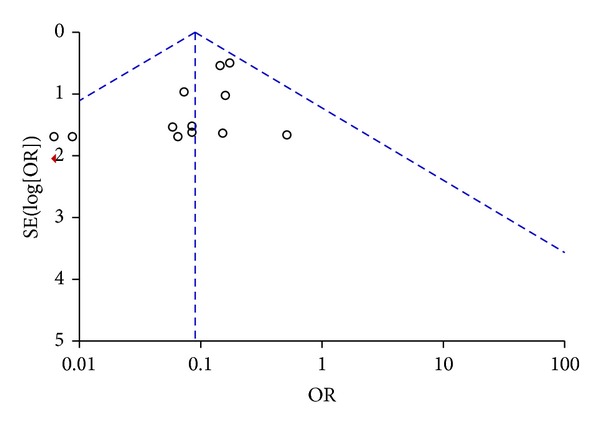
Funnel plot of comparison of AVP or terlipressin versus all other strategies (fluid resuscitation, vasoconstrictors, and placebo).

**Figure 4:**
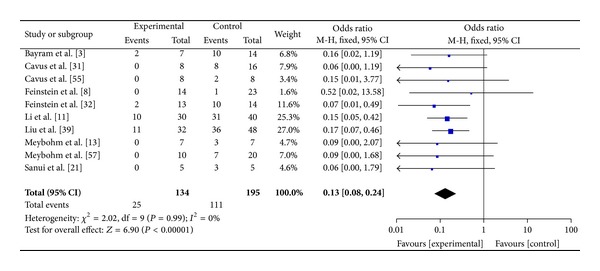
Forest plot of comparison of AVP or terlipressin versus all other strategies including studies with low risk of bias.

**Figure 5:**
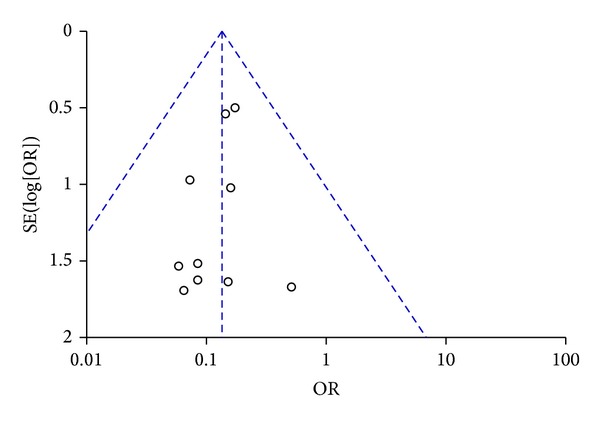
Funnel plot of comparison of AVP or terlipressin versus all other strategies including studies with low risk of bias.

Visual inspection of funnel plot identified an asymmetrical shape, suggesting the presence of publication bias (Figure 3). Sensitivity analyses performed by sequentially removing each study and reanalysing the remaining dataset (producing a new analysis for each study removed) did not lead to major changes in direction or magnitude of statistical findings. Sensitivity analyses carried out with studies with low risk of bias (eliminating the studies responsible for the asymmetry of the funnel plot) confirmed the overall results of our work showing a reduction in mortality in AVP/terlipressin animals versus controls (OR = 0.13 (95% CI 0.08–0.24); *P* for effect < 0.001, *P* for heterogeneity 0.99, *I* ^2^ = 0% with 10 studies and 329 animals included) (Figures 4 and 5). Data of mortality are summarized in Table 2.

In the majority of the studies included in this meta-analysis, AVP has been administered with an initial bolus followed by continuous infusion. Bolus doses ranged from 0.1 U/kg to 0.4 U/kg while continuous infusion dosages ranged from 0.04 U/kg/min to 0.08 U/kg/min. Other studies report AVP infusion dosages in U/kg/h that range from 0.1 [21] to 2 U/kg/h [39, 40]. In the study of Bayram et al., terlipressin was administered at the dose of 50 mcg/kg [3].

## 4. Discussion

The most important finding of this meta-analysis is that the use of AVP in the hypovolemic shock increases survival in animal studies. All studies included were randomized (AVP or terlipressin versus placebo, other vasopressors or fluid administration), were conducted on animal models (pig and rats), and were published in peer-reviewed journals.

The use of vasopressors in hypovolemic shock might contradict the conventional knowledge of how to treat this condition. Nevertheless their use in late phases of hemorrhagic shock is a common practice. Vasopressors have recently been suggested in the European guidelines for the management of hemorrhagic shock in order to maintain an adequate mean arterial pressure when fluid therapy gives no positive results [15, 41]. Guidelines recommend the use of norepinephrine as the vasopressor of choice, whilst the use of terlipressin or AVP is not mentioned.

The use of AVP and its synthetic analog terlipressin has received significant attention in clinical practice, especially in septic shock and cardiac arrest [43–46]. AVP was discovered in 1895 from the extract of the posterior pituitary gland and named after its vasoconstrictive properties [16, 42].

Landry et al. reported, for the first time, the successful administration of exogenous AVP in patients with septic shock [43]. Russell et al. compared the use of AVP versus norepinephrine in patients with septic shock in the “Vasopressin and Septic Shock Trial.”

In 779 patients the adverse effects were similar in both groups, with no differences in 28-day mortality and major organ dysfunction [44]. Another potential use of AVP is in the pharmacological treatment of cardiac arrest [45, 46]. AVP followed by epinephrine may be more effective than epinephrine alone in the treatment of refractory cardiac arrest, especially in patients with asystole [29].

In recent years, several animal studies have shown that the administration of AVP in patients with uncontrolled hemorrhagic shock is a promising treatment [10]. Our systematic analysis of literature has evaluated several clinical studies on animals. Morales et al. were the first ones to study the effects of the administration of different doses of AVP (from 1 to 4 mU/kg) in seven dogs undergoing prolonged hemorrhagic shock and concluded that AVP is an effective agent in the irreversible phase of hemorrhagic shock unresponsive to volume replacement and catecholamines [28].

For a long time the use of vasopressors in hemorrhagic shock was considered a debatable topic. During the early phases of hemorrhagic shock arterial pressure is maintained as adequate through the activation of compensatory vasoconstrictive mechanisms guaranteed by the sympathetic system that produces a venous and arterial compensatory vasoconstriction [41].

When blood loss is abundant and this mechanism is no longer efficient to maintain an adequate organ perfusion, the sympathetic system becomes inhibited with subsequent reduction of peripheral resistance and bradycardia. Hemorrhagic shock is also responsible for an abnormal vascular bed reaction mediated by nitric oxide that reduces the response to endogenous and exogenous norephineprine [47]. The trauma and organ damage developing from the shock-induced hypoperfusion bring about the activation of the inflammatory cascade with subsequent vasoplegia [48, 49].

The use of vasopressors may be helpful in these cases. In their retrospective study Plurad et al. determined that an early vasopressor exposure after a critical injury is independently associated with an increased mortality rate and this is not related to the volemic status where hypovolemic patients are those with values of central venous pressure ≤ 8 mmHg. In this retrospective study, vasopressor exposure was associated with death independent of injury severity. Vasopressor-treated patients had lower arterial pressure, required more fluids and transfusions, and had a higher serum creatine [50].

However the update of the European guidelines has recently considered the use of norepinephrine for irreversible hemorrhagic shock. There are several human case reports that have supported the use of AVP as an optimizing measure capable of supporting arterial pressure during the triage of trauma victims [27, 51].

At present, a multicenter, randomized controlled trial (Vasopressin in Traumatic Hemorrhagic Shock—VITRIS study) is being organized in Europe to evaluate the effects of AVP in prehospital management of hemorrhagic shock [52]. Unfortunately, as of now, we only have the results of retrospective studies on humans. Collier et al. conducted a retrospective cohort analysis of trauma patients requiring vasopressors within 72 hours of admission. They observed higher mortality (51% versus 41%) in patients treated with AVP concluding that its administration is associated with increased mortality in trauma patients with refractory hypotension [53]. However patients treated with AVP in this study have higher values of Trauma-Injury Severity Score (TRISS) and initial lactate levels. Arterial blood pressure values of these two groups are not reported. Grmec et al. performed a prehospital prospective cohort study to assess the influence of treatment with AVP and hydroxyethyl starch solution (HHS) on outcome in resuscitated blunt trauma patients with pulseless electrical activity (PEA) cardiac arrest. Thirty-one patients were studied concluding that victims of severe blunt trauma with PEA should be initially treated with AVP in combination with HHS for volume resuscitation followed by standard resuscitation therapy and other procedures when needed [54].

Studies conducted on animals have several limitations. Survival times measured in the experiments are different. The median value is 15.5 hours and the median is 1.5 hours. Few studies keep observing animals after six hours [11, 30]. Those studies are performed with different protocols in settings varying from head trauma [55, 56], thoracic trauma, and abdominal trauma [40] or after severe hepatic lesions [57].

Dosages used in animal trials are higher than dosages used in human studies. Humans have been successfully treated with AVP infusion of 2–4 U/h in vasodilatory shock [58, 59] and 10–20 UI boluses in patients with upper intestinal bleeding [60]. Most of the studies favorably estimate the impact of AVP to handle hemodynamic and improve survival. However it is recommended not to underestimate the possible adverse effects that might derive from the use of AVP since its use is only indicated in irreversible shock no longer treatable with fluid resuscitation alone. Vasopressin could be considered as a possible pharmacologic adjunct in patients with shock refractory to the administration of fluids and catecholamines but the use of AVP alone cannot replace the use of fluids [61]. The AVP, as well as other vasopressors, seems to be beneficial only when administered in association with fluids [62, 63].

## 5. Conclusions

Data acquired from our meta-analysis suggest strong scientific evidence for the efficacy of AVP for the early treatment of hemorrhagic shock in animal models. AVP has shown to be more effective than all other treatments, including other vasopressors drugs. We are awaiting the results of the VITRIS [50] study to confirm in humans the results obtained in animal studies.

## 6. Methodological Limitations

The purposes, designs, and conduct are different between systematic review and meta-analysis of preclinical and clinical studies. Clinical reviews are intrinsically confirmatory and the aim of a Cochrane review is to provide evidence to allow practitioners and patients to make informed decisions about the delivery of health-care. Animal studies are meant to be exploratory and do not lead to definitive conclusions directly applicable to humans [64].

The results shown should be interpreted with caution. Animal studies are inherently heterogeneous, more than the typical clinical trials. Successfully translating findings to human diseases depends largely upon understanding the sources of heterogeneity and their impact on effect size [64]. The study is conducted without randomized controlled trials in humans, and our findings should only be considered as a* hypothetical suggestion* for further research, awaiting the results of randomized controlled human trials.

## Supplementary Material

In the supplemental materials we confronted AVP/terlipressin with different comparators singularly: fluid resuscitation (fig. 6a), placebo (6b), other vasoconstrictive drugs(6C) and norepineprhine(6d). In all the analysis we conducted AVP/terlipressin was associated to a reduction of the death rate. We also did a meta-analysis on survival considering separately the studies conducted on rats (fig. 7a) and on pigs (fig.7b). In fig.8 we considered only the studies where hemorrhagic shock was due to a splancnic bleeding. We then did a meta-analysis excluding those trials with zero mortality (fig. 9) and selecting the studies that had mortality as the primary end-point. In table 3 are reported the dosages of AVP, terlipressin, vasopressors and the total amount of fluids included in the studies in the meta-analysis. In table 4 are reported the primary end-points and the setting of the included studies.

## Figures and Tables

**Table 1:** Studies included in the meta-analysis.

1st author	Journal	Year	Number of AVP (V) or terlipressin (T)	Number of Controls	Control	Animal
Bayram [3]	Am J Emerg Med	2012	7 (T)	14	Placebo (7); Ringer lactate (7)	Rats

Cavus [31]	Resuscitation	2009	8 (V)	8	Fluid resuscitation (8)	Pigs

Cavus [55]	Resuscitation	2010	8 (V)	16	Fluid resuscitation (8); noradrenaline + HS (8)	Pigs

Dudkiewicz [56]	Crit Care Med	2008	10 (V)	10	Phenylephrine (10)	Pigs

Feinstein [8]	J Am Coll Surg	2005	14 (V)	23	Crystalloid (9); phenylephrine (5); crystalloid + phenylephrine (9)	Pigs

Feinstein [32]	J Trauma	2005	8 (V)	9	NS (9)	Pigs

Li [11]	J Surg Res	2011	30 (V)	40	Placebo (10); Ringer lactate (10); whole blood (10); NE (10)	Rats

Liu [39]	Shock	2013	32 (V)	48	Hypotensive resuscitation (16); Ringer lactate (16); NE (16)	Rats

Meybohm [13]	J Trauma	2007	7 (V)	7	HHS + NE (7)	Pigs

Meybohm [57]	Resuscitation	2008	10 (V)	20	Fluid (10); HHS + NS (10)	Pigs

Raedler [10]	Anesth Analg	2004	7 (V)	14	Saline placebo (7); fluid resuscitation (7)	Pigs

Sanui [21]	Crit Care Med	2006	5 (V)	5	Placebo (5)	Pigs

Stadlbauer [30]	Anesthesiology	2003	9 (V)	14	Saline placebo (7); fluid resuscitation (7)	Pigs

Stadlbauer [40]	Crit Care	2007	7 (V)	12	Saline placebo (5); fluid resuscitation (7)	Pigs

Voelckel [1]	Crit Care Med	2003	7 (V)	14	Epinephrine (7); saline placebo (7)	Pigs

**Table 2:** Results for mortality.

Outcome	Number of included trials	AVP/terlipressin animals	Control animals	OR	95% CI	*P* for effect	*P* for heterogeneity	*I* ^2^ (%)
Overall trials	15	174	259	0.09	0.05–0.15	<0.001	0.30	14
Mortality		15%	63%					

Placebo as comparator drug	7	72	48	0.03	0.01–0.09	<0.001	0.57	0
Mortality		18%	92%					

Fluid resuscitation as comparator drug	11	114	117	0.08	0.04–0.15	<0.001	0.75	0
Mortality		18%	67%					

Vasopressors (NE or epinephrine) as comparator drug	7	88	87	0.18	0.08–0.44	<0.001	0.96	0
Mortality		18%	39%					

NE as comparator drug	4	54	53	0.16	0.06–0.45	<0.001	0.97	0
Mortality		20%	47%					

Sensitivity analysis (including only low risk of bias studies)	10	134	195	0.13	0.08–0.24	<0.001	0.99	0
Mortality		18%	57%					

## Appendix

(“Vasopressin”[MeSH Terms] OR terlipressin[Text Word] OR “arginine vasopressin”[Text Word]) AND (“hemorrhagic shock” OR trauma) AND ((randomized controlled trial[pt] OR controlled clinical trial[pt] OR randomized controlled trials[mh] OR random allocation[mh] OR double-blind method[mh] OR single-blind method[mh] OR clinical trial[pt] OR clinical trials[mh] OR (“clinical trial”[tw] OR ((singl∗[tw] OR doubl∗[tw] OR trebl∗[tw] OR tripl∗[tw]) AND (mask∗[tw] OR blind[tw])) OR (“latin square”[tw]) OR placebos[mh] OR placebo∗[tw] OR random∗[tw] OR research design[mh:noexp] OR comparative study[mh] OR evaluation studies[mh] OR follow-up studies[mh] OR prospective studies[mh] OR crossover studies[mh] OR control∗[tw] OR prospective∗[tw] OR volunteer∗[tw]) OR (animal[mh] OR human[mh]) NOT (comment[pt] OR editorial[pt] OR (meta-analysis[pt] NOT clinical trial[pt]) OR practice-guideline[pt] OR review[pt]))).
