# Supplementary material for: Vasopressin in Hemorrhagic Shock: A Systematic Review and Meta-Analysis of Randomized Animal Trials
Source: Biomed Res Int. 2014 Sep 1;2014:421291. doi: 10.1155/2014/421291 (PMC4165559; doi:10.1155/2014/421291)
Supplement: Supplementary file 1 — In the supplemental materials we confronted AVP/terlipressin with different comparators singularly: fluid resuscitation (fig. 6a), placebo (6b), other vasoconstrictive drugs(6C) and norepineprhine(6d). In all the analysis we conducted AVP/terlipressin was associated to a reduction of the death rate. We also did a meta-analysis on survival considering separately the studies conducted on rats (fig. 7a) and on pigs (fig.7b). In fig.8 we considered only the studies where hemorrhagic shock was due to a splancnic bleeding. We then did a meta-analysis excluding those trials with zero mortality (fig. 9) and selecting the studies that had mortality as the primary end-point. In table 3 are reported the dosages of AVP, terlipressin, vasopressors and the total amount of fluids included in the studies in the meta-analysis. In table 4 are reported the primary end-points and the setting of the included studies. [file 421291.f1.zip › supp/1038648.pdf]

Vasopressin in hemorrhagic shock: a systematic review and meta-analysis of randomized animal trials.

Cossu AP[1], Mura P[1], De Giudici LM[1], Puddu D[1], Pasin L[2], Evangelista M[3], Xanthos T[4], Musu M[1] and Finco G[1].

[1] UOC Anesthesia and Intensive Care and, Pain Therapy Service, AOU Cagliari
Department of Medical Sciences “M. Aresu”, University of Cagliari, Italy.

[2] Department of Anesthesia and Intensive Care, Vita-Salute San Raffaele University, Milan, Italy.

[3] Department of Anesthesia and Intensive Care, Catholic University, Rome, Italy

[4] MSc Program “Cardiopulmonary Resuscitation”, University of Athens, Medical School, Hellenic Society of Cardiopulmonary Resuscitation.

Corresponding Author: Cossu Andrea Pasquale. andreapcossu@yahoo.it.

Keywords: vasopressin, terlipressin, haemorrhagic shock, hypovolemic shock, vasopressors.

Abstract

Objective: The latest European guidelines for the management of hemorrhagic shock suggest the use of vasopressors (norepinephrine) in order to restore an adequate mean arterial pressure when fluid resuscitation therapy fails to restore blood pressure. The administration of arginine vasopressin (AVP), or its analogue terlipressin, has been proposed as an alternative treatment in the early stages of hypovolemic shock. *Design:* A meta-analysis of randomized-controlled animal trials.

Participants: A total of 433 animals from 15 studies were included in the analysis.

Interventions: The ability of AVP and terlipressin to reduce mortality when compared with fluid resuscitation therapy, other vasopressors (norepinephrine or epinephrine) or placebo was investigated.

Measurements and Main Results: Google Scholar and PubMed were searched (updated November 4th, 2013). Authors and external experts were contacted. Pooled estimates showed that AVP and terlipressin consistently and significantly improve survival in hemorrhagic shock (mortality: 26/174 [15%] in the AVP group vs 164/259 [63%] in the control arms; OR = 0.09; 95% CI 0.05 to 0.15; p for effect < 0.001; p for heterogeneity=0.30; I^2 = 14%).

Conclusions: Results suggest that AVP and terlipressin improve survival in the early phases of animal models of hemorrhagic shock. Vasopressin seems to be more effective than all other treatments, including other vasopressor drugs. These results need to be confirmed by human randomized clinical trials.

Introduction

Trauma is the principal cause of death for people under 35 years of age, with more than 5 million injury-related deaths every year in the world. Approximately 30% of these deaths can be attributed to hemorrhagic shock.[1,2] Untreated prehospital hemorrhagic shock is one of the leading causes of cardiac arrest.[3,4] Appropriate management and treatment are necessary to prevent adverse events and outcomes.[5,6,7] The early phase of hemorrhagic shock is characterized by a vasoconstrictive response and if the shock is left untreated it can lead to vasodilation that doesn't respond to conventional resuscitation strategies.[8, 9] Pre-hospital hemorrhagic shock treatment should be focused on maintaining adequate mean arterial pressure (MAP) along with organ perfusion up until arrival at the hospital.[10,11]

Small volume resuscitation with colloids or hyperoncotic fluids may be useful during early phases of uncontrolled bleeding[12,13,14]. Recent international guidelines suggest that vasopressors may also be required to maintain tissue perfusion where fluid resuscitation itself doesn't achieve the expected goal[15].

Arginine vasopressin (AVP) is an endogenous neurohypophysial hormone with an antidiuretic function. The most important AVP release stimulus is the plasma osmolality variation followed by blood pressure variations.[16,17,18] AVP also suppresses nitric oxide (NO) production.[19] The AVP release may also be suppressed by increased levels of norepinephrine and the increased release of NO from vascular endothelium of the posterior pituitary gland.[20,21]Terlipressin is a long-acting synthetic analogue of AVP, proposed in the septic shock management as a rescue therapy, when adequate MAP values are difficult to reach with standard therapy. It's characterized by a longer duration of action and a higher selectivity on the V_1 receptors that limits the edemigenous effect mediated by the V_2 receptors differently from what its native counterpart does.[22] AVP or

terlipressin can be both used with the aim to reach the desired MAP target or to reduce the norepinephrine dosage.[23, 24]

In animal models in which severe uncontrolled blood loss has been induced, the administration of AVP has shown improvement in survival, neurologic outcome and enhanced hemodynamic performance.[25,26,27] During the irreversible phase of hemorrhagic shock, unresponsive to fluids and catecholamines administration, AVP can mediate peripheral vasoconstriction through V_1 receptors.[13, 28, 29] AVP works primarily on arterioles in extracerebral tissues, with less constriction action on coronary and renal vessels with potential vasodilatory effect on cerebral and pulmonary flow.[30] Recent animal studies have shown that AVP treatment can achieve hemodynamic optimization during pre-hospital hemorrhagic shock, while fluids and catecholamines showed neither improvement of hemodynamic parameters nor survival.[1,31, 32]

AVP use is associated with some adverse effects such as ischemic complications especially in cardiac, splanchnic and skin circulation.[33] The decreased gut perfusion may determine tissue necrosis with subsequent translocation of bacteria that promotes the development of sepsis in the post-resuscitation phase.[34] The increased expression of the V_1 receptor subtype in trauma brain injury might promote the development of cerebral edema.[8,35]

To evaluate the impact on survival of V_1 receptor agonists in hypovolemic refractory shock, we conducted a systematic review and meta-analysis of data pooled from existing trials comparing AVP or terlipressin and conventional shock management in mammals.

Materials and methods

Search Strategy

All randomized animal trials using AVP or terlipressin in hypovolemic shock were identified. Relevant studies were independently searched by two trained investigators in

Google Scholar and PubMed (updated November 4th, 2013). The full PubMed search strategy, including as keywords AVP, arginine-vasopressin, terlipressin, hemorrhagic and hypovolemic shock was developed according to Biondi Zoccai et al. and is available in Appendix 1.[36]

Study Selection

References obtained from databases and literature searches were first examined independently at the title/abstract level by two investigators, with divergences resolved by consensus and then, if potentially pertinent, retrieved as a complete article.

Inclusion criteria for potentially relevant studies were: random allocation to treatment; animal experimental design; comparison of AVP or terlipressin (with or without fluid administration) versus placebo or fluids or catecholamines or both fluids plus catecholamines. Exclusion criteria were: duplicate publications, human trials, studies with no data on survival. Two investigators selected studies for the final analysis by independently assessing compliance to the selection criteria. Divergences from the selection criteria were resolved by consensus.

Data Abstraction and Study Characteristics

Two investigators independently extracted data on the study design, experimental setting, dosages of AVP or terlipressin, experimental duration, with divergences resolved by consensus. If the required data could not be retrieved from the published report, at least two separate attempts to contact the original authors were made.

The primary end-point was mortality at the longest available follow-up. In addition, we performed further subanalysis comparing animals treated with AVP (or terlipressin) with those treated respectively with placebo, fluid resuscitation and other vasoconstrictive drugs.

Data Analysis and Synthesis

Computations were performed with RevMan 4.2.[35] Binary outcomes from individual studies were analyzed to compute individual odds ratios (ORs) with pertinent 95% confidence intervals (CIs), and a pooled summary effect estimate was calculated by means of the Mantel-Haenszel method and the fixed effect-model in case of low statistical inconsistency (I^2 <25%) or the random-effect model in case of moderate or high statistical inconsistency (I^2>25%).[37] Statistical heterogeneity and inconsistency were measured using Cochran Q tests and I^2 (by Higgins and Thompson), respectively.[38] Statistical significance was set at 2-tailed 0.05 for hypothesis testing and at 0.10 for heterogeneity testing. According to Higgins et al., the I^2 values around 25%, 50%, and 75% were considered to represent respectively low, moderate and severe statistical inconsistency.[38] The risk of publication bias was assessed by visual inspection of the funnel plot for mortality. Sensitivity analyses were performed by sequentially removing each study and reanalysing the remaining dataset (producing a new analysis for each study removed) and by analysing only data from studies with low risk of bias.

Results

Study Characteristics

Database searches, backwards snowballing and contacts with experts yielded a total of 246 citations. After excluding non-pertinent titles or abstracts, 22 studies were retrieved in complete form and assessed according to the selection criteria (Fig 1). Seven studies were further excluded for the absence of survival data. Fifteen eligible trials were included in the final analysis.

The 15 included studies randomized 433 animals, 174 to AVP (14 trials) or terlipressin (one trial) and 259 to control (placebo, vasopressors or fluid resuscitation). The included trials were conducted on pigs (12 studies) and on rats (three studies). All

manuscripts were published in indexed journals. Detailed study characteristics are summarized in table 1.

Quantitative Data Synthesis

The overall analysis showed that AVP/terlipressin were associated with a reduction in animal mortality (26/174 [15%] in the AVP/terlipressin group vs 164/259 [63%] in the control arms; OR = 0.09 [95% CI 0.05-0.15]; p for effect < 0.001; p for heterogeneity=0.30; I^2 = 14%) (Fig. 2). When studies were grouped to either fluid resuscitation, placebo, norepinephrine or other vasoconstrictive drug as a comparator, administration of AVP/terlipressin was still associated with a reduction in mortality. (Supplemental figures 6 b-e).

Visual inspection of funnel plot identified an asymmetrical shape, suggesting the presence of publication bias (Fig. 3). Sensitivity analyses performed by sequentially removing each study and reanalysing the remaining dataset (producing a new analysis for each study removed), did not lead to major changes in direction or magnitude of statistical findings. Sensitivity analyses carried out with studies with low risk of bias (eliminating the studies responsible for the asymmetry of the funnel plot) confirmed the overall results of our work showing a reduction in mortality in AVP/terlipressin animals versus controls (OR= 0.13 [95% CI 0.08-0.24]; p for effect < 0.001, p for heterogeneity 0.99, I^2= 0% with 10 studies and 329 animals included). (Fig. 4,5).

In the majority of the studies included in this meta-analysis, AVP has been administered with an initial bolus followed by continuous infusion. Bolus doses ranged from 0.1U/kg to 0.4U/kg while continuous infusion dosages from 0.04U/kg/min to 0.08U/kg/min. Other studies report AVP infusion dosages in U/kg/h that range from 0.1[21] to 2U/kg/h[39,40] In the study of Bayram B et al, terlipressin was administered at the dose of 50mcg/kg.[3]

Discussion

The most important finding of this meta-analysis is that the use of AVP in the hypovolemic shock increases survival in animal studies. All studies included were randomized (AVP or terlipressin versus placebo, other vasopressors or fluid administration), were conducted on animal models (pig and rats) and were published in peer-reviewed journals.

The use of vasopressors in hypovolemic shock might contradict the conventional knowledge of how to treat this condition. Nevertheless their use in late phases of hemorrhagic shock is a common practice. Vasopressors have recently been suggested in the European guidelines for the management of hemorrhagic shock in order to maintain an adequate mean arterial pressure when fluid therapy gives no positive results.[15,41] Guidelines recommend the use of norepinephrine as the vasopressor of choice, whilst the use of terlipressin or AVP is not mentioned.

The use of AVP, and its synthetic analog terlipressin, has received significant attention in clinical practice, especially in septic shock and cardiac arrest.[43, 44, 45, 46] AVP was discovered in 1895 from the extract of the posterior pituitary gland and named after its vasoconstrictive properties.[16,42]

Landry *et al* reported for the first time, the successful administration of exogenous AVP in patients with septic shock.[43] Russell *et al* compared the use of AVP versus norepinephrine in patients with septic shock in the "Vasopressin and Septic Shock Trial".

In 779 patients the adverse effects were similar in both groups, with no differences in 28-day mortality and major organ dysfunction.[44] Another potential use of AVP is in the pharmacological treatment of cardiac arrest.[45,46] AVP followed by epinephrine may be more effective than epinephrine alone in the treatment of refractory cardiac arrest, especially in patients with asystole.[29]

In recent years, several animal studies have shown that the administration of AVP in patients with uncontrolled hemorrhagic shock is a promising treatment.[10] Our systematic analysis of literature has evaluated several clinical studies on animals. Morales *et al* were the first ones to study the effects of the administration of different doses of AVP (from 1 to 4 mU/kg) in seven dogs undergoing prolonged hemorrhagic shock and concluded that AVP is an effective agent in the irreversible phase of hemorrhagic shock unresponsive to volume replacement and catecholamines.[28]

For a long time the use of vasopressors in hemorrhagic shock was considered a debatable topic. During the early phases of hemorrhagic shock arterial pressure is maintained adequate through the activation of compensatory vasoconstrictive mechanisms guaranteed by the sympathetic system that produces a venous and arterial compensatory vasoconstriction. [41]

When blood loss is abundant and this mechanism is no longer efficient to maintain an adequate organ perfusion, the sympathetic system becomes inhibited with subsequent reduction of peripheral resistance and bradycardia. Hemorrhagic shock is also responsible for an abnormal vascular bed reaction mediated by nitric oxide that reduces the response to endogenous and exogenous norephineprine.[47] The trauma and organ damage developing from the shock-induced hypoperfusion brings about the activation of the inflammatory cascade with subsequent vasoplegia. [48,49]

The use of vasopressors may be helpful in these cases. In their retrospective study Plurad *et al*. determined that an early vasopressor exposure after a critical injury is independently associated with an increased mortality rate and this is not related to the volemic status where hypovolemic patients are those with values of central venous pressure $\leq$ 8 mmHg. In this retrospective study, vasopressor exposure was associated with death independent of injury severity. Vasopressor-treated patients had lower arterial pressure, required more fluids and transfusions and had a higher serum creatine.[50]

However the update of the European guidelines has recently considered the use of norepinephrine for irreversible hemorrhagic shock. There are several human case reports that have supported the use of AVP as an optimizing measure capable to support arterial pressure during the triage of trauma victims.[27,51]

At present, a multicenter, randomized controlled trial (Vasopressin in Traumatic Hemorrhagic Shock – VITRIS study) is being organized in Europe to evaluate the effects of AVP in prehospital management of hemorrhagic shock.[52] Unfortunately, as of now, we only have the results of retrospective studies on humans. Collier *et al* conducted a retrospective cohort analysis of trauma patients requiring vasopressors within 72 hours of admission. They observed higher mortality (51% vs 41%) in patients treated with AVP concluding that its administration is associated with increased mortality in trauma patients with refractory hypotension.[53] However patients treated with AVP in this study have higher values of Trauma - Injury Severity Score (TRISS) and initial lactate levels. Arterial blood pressure values of these two groups are not reported. Grmec *et al* performed a prehospital prospective cohort study to assess the influence of treatment with AVP and hydroxyethyl starch solution (HHS) on outcome in resuscitated blunt trauma patients with pulseless electrical activity (PEA) cardiac arrest. Thirty-one patients were studied concluding that victims of severe blunt trauma with PEA should be initially treated with AVP in combination with HHS for volume resuscitation followed by standard resuscitation therapy and other procedures when needed.[54]

Studies conducted on animals have several limitations. Survival times measured in the experiments are different. The median value is 15.5 hours and the median is 1.5 hours. Few studies keep observing animals after six hours[11,30] Those studies are performed with different protocols in settings varying from head trauma[55,56],thoracic trauma, abdominal trauma[40] or after severe hepatic lesions[57].

Dosages used in animal trials are higher than dosages used in human studies. Humans have been successfully treated with AVP infusion of 2-4U/h in vasodilatory shock [58,59] and 10-20UI boluses in patients with upper intestinal bleeding.[60] Most of the studies favorably estimate the impact of AVP to handle hemodynamic and improve survival. However it is recommended not to underestimate the possible adverse effects that might derive from the use of AVP since its use is only indicated in irreversible shock no longer treatable with fluid resuscitation alone. Vasopressin could be considered as a possible pharmacologic adjunct in patients with shock refractory to the administration of fluids and catecholamines but the use of AVP alone cannot replace the use of fluids.[61] The AVP, as well as other vasopressors, seems to be beneficial only when administered in association with fluids.[62,63]

Conclusions

Data acquired from our meta-analysis suggest strong scientific evidence for the efficacy of AVP for the early treatment of hemorrhagic shock in animal models. AVP have shown to be more effective than all other treatments, including other vasopressors drugs. We are awaiting the results of the VITRIS[50] study to confirm in humans the results obtained in animal studies.

Methodological limitations

The purposes, designs and conduct are different between systematic review and meta-analysis of preclinical and clinical studies. Clinical reviews are intrinsically confirmatory and the aim of a Cochrane review is to provide evidence to allow practitioners and patients to make informed decisions about the delivery of health-care. Animal studies are meant to be exploratory and do not lead to definitive conclusions directly applicable to humans.[64]

The results shown should be interpreted with caution. Animal studies are inherently heterogeneous, and more so than atypical clinical trial. Successfully translating findings to human diseases depends largely upon understanding the sources of heterogeneity, and their impact on effect size.[64] The study is conducted without randomized controlled trials in humans, and our findings should only be considered as an *hypothetical suggestion* for further research, awaiting the results of randomized controlled human trials.

APPENDIX

("vasopressin"[MeSH Terms] OR terlipressin[Text Word] OR "arginine vasopressin"[Text Word]) AND ("hemorrhagic shock" OR trauma) AND ((randomized controlled trial[pt] OR controlled clinical trial[pt] OR randomized controlled trials[mh] OR random allocation[mh] OR double-blind method[mh] OR single-blind method[mh] OR clinical trial[pt] OR clinical trials[mh] OR ("clinical trial"[tw] OR ((singl*[tw] OR doubl*[tw] OR trebl*[tw] OR tripl*[tw]) AND (mask*[tw] OR blind[tw])) OR ("latin square"[tw]) OR placebos[mh] OR placebo*[tw] OR random*[tw] OR research design[mh:noexp] OR comparative study[mh] OR evaluation studies[mh] OR follow-up studies[mh] OR prospective studies[mh] OR crossover studies[mh] OR control*[tw] OR prospectiv*[tw] OR volunteer*[tw]) OR (animal[mh] OR human[mh]) NOT (comment[pt] OR editorial[pt] OR (meta-analysis[pt] NOT clinical trial[pt]) OR practice-guideline[pt] OR review[pt])))

Acknowledgements: We are indebted to Toby Schwartzbarth for the revision of the manuscript.

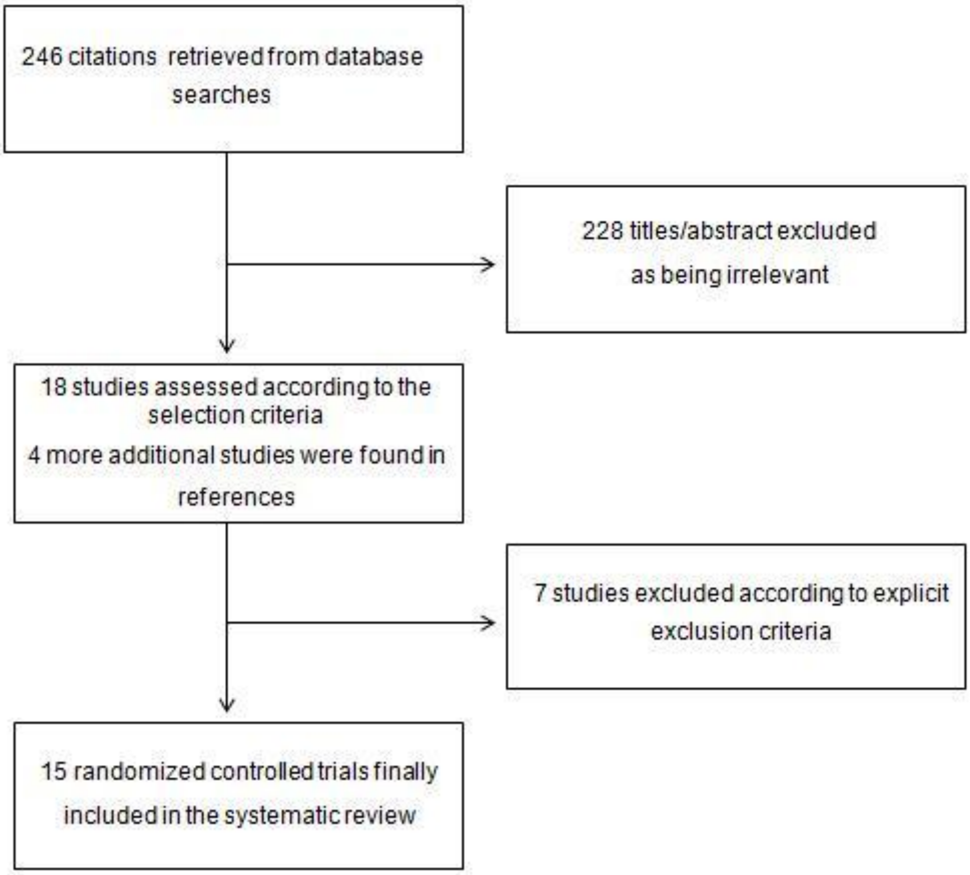

Fig 1. Flow diagram of the systematic review process

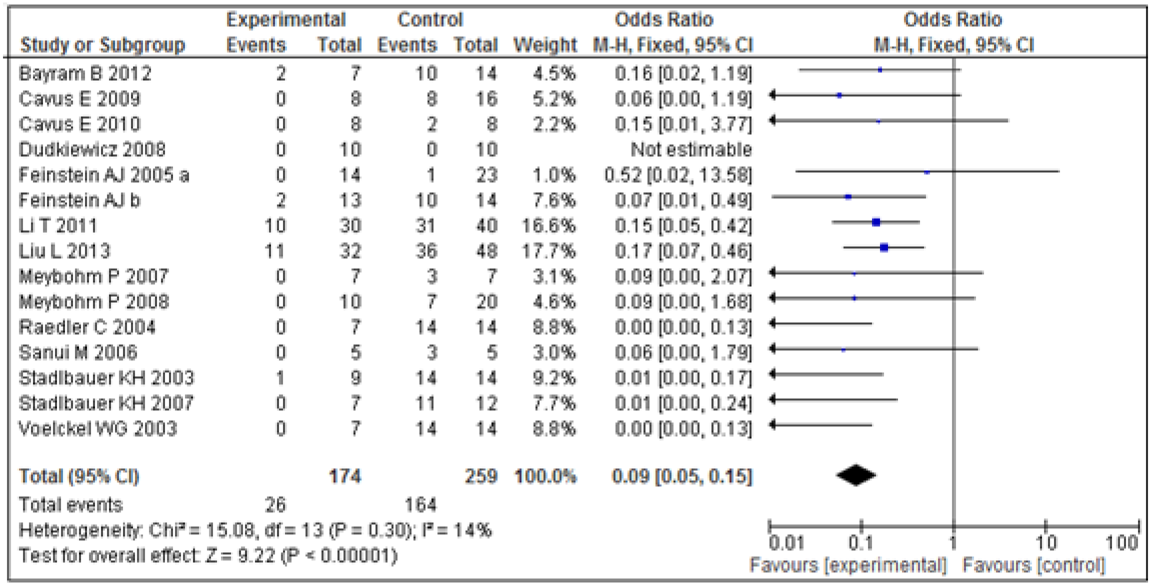

Fig 2: AVP or terlipressin vs all other strategies (fluid resuscitation, vasoconstrictors, placebo)

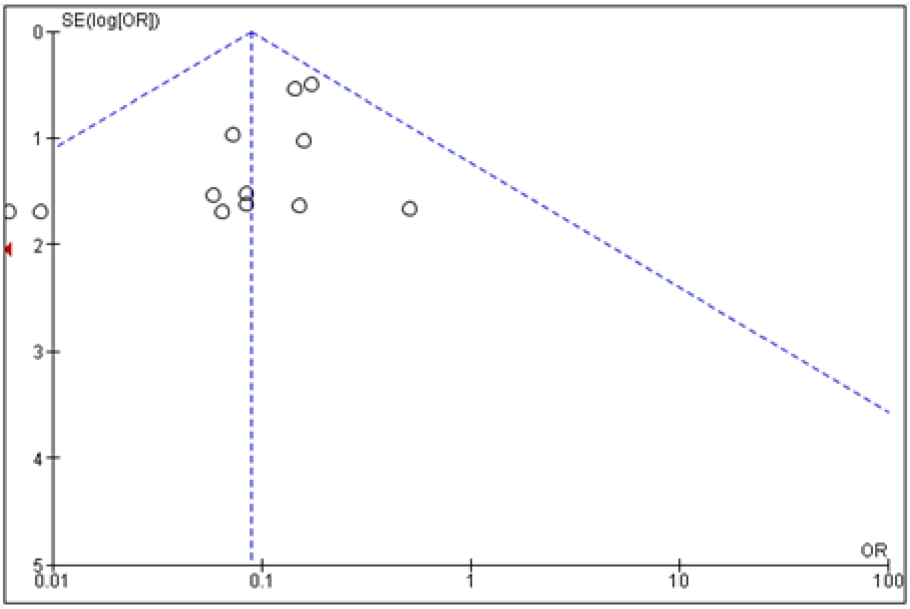

Fig 3: Funnel plot of comparison of AVP or terlipressin vs all other strategies (fluid resuscitation, vasoconstrictors, placebo)

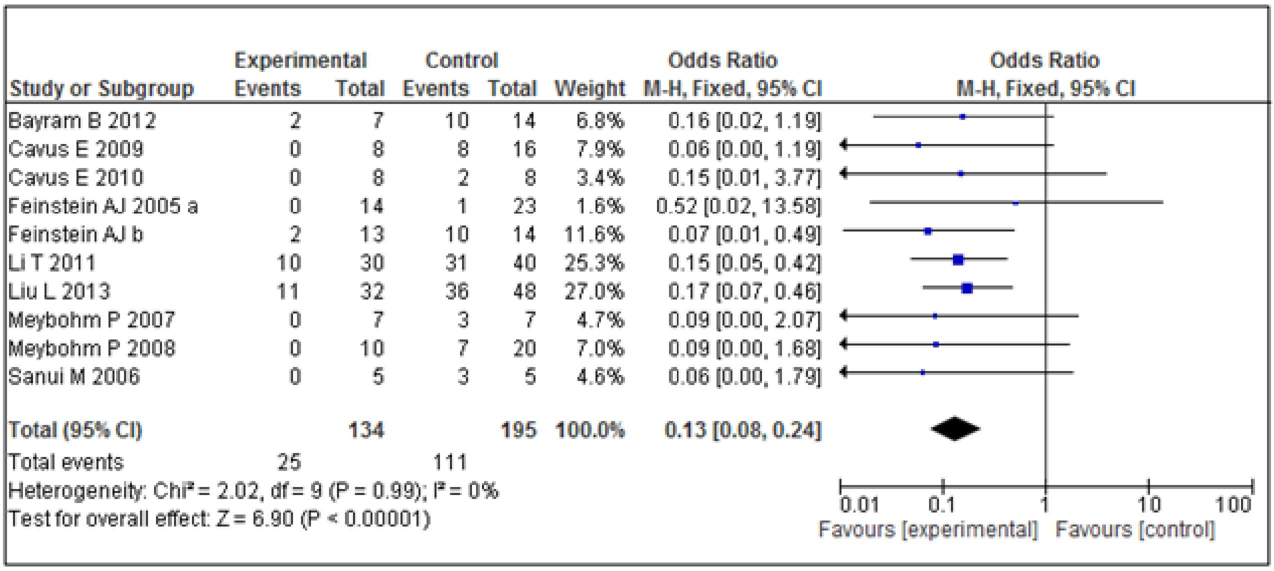

Fig 4: Forest plot of comparison of AVP or terlipressin vs all other strategies including studies with low risk of bias

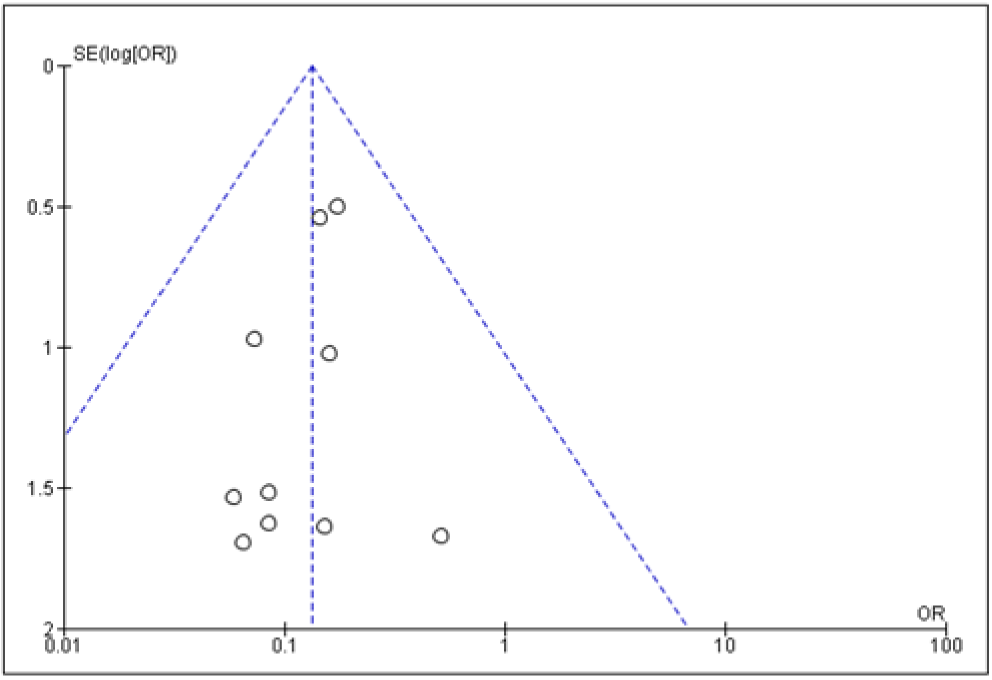

Fig 5: Funnel plot of comparison of AVP or terlipressin vs all other strategies including studies with low risk of bias

1st Author	Journal	Year	N° AVP(V) or Terlipressin(T)	N° Control	Control	Animal
**Bayram B** [3]	Am J Emerg Med	2012	7 (T)	14	Placebo (7); Ringer Lactate (7)	Rats
**Cavus E** [31]	Resuscitation	2010	8 (V)	8	Fluid resuscitation (8)	Pigs
**Cavus E** [55]	Resuscitation	2009	8 (V)	16	Fluid resuscitation (8); Noradrenaline + HS (8)	Pigs
**Dudkiewicz M** [56]	Crit Care Med	2008	10 (V)	10	Phenylephrine (10)	Pigs
**Feinstein AJ** [8]	J Am Coll Surg	2005	14 (V)	23	Crystalloid (9); Phenylephrine(5); Crystalloid + phenylephrine(9)	Pigs
**Feinstein AJ** [32]	J Trauma	2005	8 (V)	9	NS (9)	Pigs
**Li T** [11]	J Surg Res	2011	30 (V)	40	Placebo (10); Ringer Lactate (10); Whole blood (10);NE (10)	Rats
**Liu L** [39]	Shock	2013	32 (V)	48	Hypotensive resuscitation (16); Ringer Lactate (16); NE (16)	Rats
**Meybohm P** [13]	J Trauma	2007	7 (V)	7	HHS + NE (7)	Pigs
**Meybohm P** [57]	Resuscitation	2008	10 (V)	20	Fluid (10); HHS+NS (10)	Pigs
**Raedler C** [10]	Anesth Analg	2004	7 (V)	14	Saline placebo (7); Fluid resuscitation (7)	Pigs
**Sanui M** [21]	Crit Care Med	2006	5 (V)	5	Placebo (5)	Pigs
**Stadlbauer KH** [30]	Anesthesiology	2003	9 (V)	14	Saline placebo (7); Fluid resuscitation (7)	Pigs
**Stadlbauer KH** [40]	Crit Care	2007	7 (V)	12	Saline placebo (5); Fluid resuscitation (7)	Pigs
**Voelckel WG** [1]	Crit Care Med	2003	7 (V)	14	Epinephrine (7); Saline placebo (7)	Pigs

Table 1: studies included in the meta-analysis

Outcome	Number of included trials	AVP/terlipressin animals	Control animals	OR	95% CI	P for effect	P for heterogeneity	I2 (%)
**Overall trials**	15	174	259	0.09	0.05-0.15	< 0.001	0.30	14
**Mortality**		15%	63%					
**Placebo as comparator drug**	7	72	48	0.03	0.01-0.09	< 0.001	0.57	0
**Mortality**		18%	92%					
**Fluid resuscitation as comparator drug**	11	114	117	0.08	0.04-0.15	< 0.001	0.75	0
**Mortality**		18%	67%					
**Vasopressors (NE or epinephrine) as comparator drug**	7	88	87	0.18	0.08-0.44	< 0.001	0.96	0
**Mortality**		18%	39%					
**NE as comparator drug**	4	54	53	0.16	0.06-0.45	< 0.001	0.97	0
**Mortality**		20%	47%					
**SENSITIVITY ANALYSIS** (including only low risk of bias studies)	10	134	195	0.13	0.08-0.24	< 0.001	0.99	0
**Mortality**		18%	57%					

Table 2: Results for mortality

References

1. Voelckel, W.G., Raedler, C., Wenzel, V., Lindner, K.H., Krismer, A.C., Schmittinger, C.A., Herff, H., Rheinberger, K., Konigsrainer, A. Arginine vasopressin, but not epinephrine, improves survival in uncontrolled hemorrhagic shock after liver trauma in pigs. Crit Care Med 2003;31:1286–1287.

2. Shoemaker WC, Peitzman AB, Bellamy R, et al: Resuscitation from severe hemorrhage. Crit Care Med 1996; 24:S12–S23

3. Bayram B, Hocaoglu N, Atilla R, Kalkan S. Effects of terlipressin in a rat model of severe uncontrolled hemorrhage via liver injury. Am J Emerg Med. 2012 Sep;30(7):1176-82.

4. Santry HP, Alam HB. Fluid resuscitation: past, present, and the future. Shock 2010;33(3):229-41.

5. Cheung AT, To PL, Chan DM, Ramanujam S, Barbosa MA, Chen PC, Driessen B, Jahr JS, Gunther RA. Comparison of treatment modalities for hemorrhagic shock. Artif Cells Blood Substit Immobil Biotechnol. 2007;35(2):173-90.

6. Stadlbauer KH, Wagner-Berger HG, Wenzel V, Voelckel WG, Krismer AC, Klima, G, Rheinberger K, Pehlaner S, Mayr VD, Lindner KH. Survival with full neurologic recovery after prolonged cardiopulmonary resuscitation with a combination of vasopressin and epinephrine in pigs. Anesth Analges 2003;96:1743–1749.

7. Bonanno FG. Hemorrhagic shock: The "physiology approach". J Emerg Trauma Shock. 2012;5(4):285-95.

8. Feinstein AJ, Patel MB, Sanui M, Cohn SM, Majetschak M, Proctor KG. Resuscitation with pressors after traumatic brain injury. J Am Coll Surg. 2005;201(4):536-45.

9. Fang Y, Li T, Fan X, Zhu Y, Liu L. Beneficial effects of activation of PKC on hemorrhagic shock in rats. J Trauma. 2010;68(4):865-73.

10. Raedler C, Voelckel WG, Wenzel V, Krismer AC, Schmittinger CA, Herff H, Mayr VD, Stadlbauer KH, Lindner KH, Königsrainer A. Treatment of uncontrolled hemorrhagic shock after liver trauma: fatal effects of fluid resuscitation versus improved outcome after vasopressin. Anesth Analg. 2004;98(6):1759-66.

11. Li T, Fang Y, Zhu Y, Fan X, Liao Z, Chen F, Liu L. A small dose of arginine vasopressin in combination with norepinephrine is a good early treatment for uncontrolled hemorrhagic shock after hemostasis. J Surg Res. 2011;169(1):76-84.

12. Bickell WH, Wall MJ, Jr., Pepe PE, et al. Immediate versus delayed fluid resuscitation for hypotensive patients with penetrating torso injuries. N Engl J Med. 1994;331:1105–1109.

13. Meybohm P, Cavus E, Bein B, Steinfath M, Weber B, Hamann C, Scholz J, Dörges V. Small volume resuscitation: a randomized controlled trial with either norepinephrine or vasopressin during severe hemorrhage. J Trauma. 2007 Mar;62(3):640-6.

14. Voelckel WG, Convertino VA, Lurie KG, et al. Vasopressin for hemorrhagic shock management: revisiting the potential value in civilian and combat casualty care. J Trauma 2010;69(Suppl 1):S69-74.

15. Spahn DR, Bouillon B, Cerny V, Coats TJ, Duranteau J, Fernández-Mondéjar E, Filipescu D, Hunt BJ, Komadina R, Nardi G, Neugebauer E, Ozier Y, Riddez L, Schultz A, Vincent JL, Rossaint R. Management of bleeding and coagulopathy following major trauma: an updated European guideline. Crit Care. 2013 Apr 19;17(2):R76. [Epub ahead of print]

16. Holt NF, Haspel KL. Vasopressin: A Review of Therapeutic Applications. J Cardiothorac Vasc Anesth. 2010 Apr;24(2):330-47.

17. Bankir L. Antidiuretic action of vasopressin: quantitative aspects and interaction between V1a and V2 receptor-mediated effects. Cardiovasc Res. 2001;51(3):372-90.

18. Vincent JL, Su F. Physiology and pathophysiology of the vasopressinergic system. Best Pract Res Clin Anaesthesiol. 2008;22(2):243-52.

19. Moreau R, Barriere E, Tazi KA, et al: Terlipressin inhibits in vivo aortic iNOS expression induced by lipopolysaccharide in rats with biliary cirrhosis. Hepatology 36:1070-1078, 2002.

20. Wenzel V, Lindner KH: Employing vasopressin during cardiopulmonary resuscitation and vasodilatory shock as a lifesaving vasopressor. Cardiovasc Res 2001;51:529–41.

21. Sanui M, King DR, Feinstein AJ, Varon AJ, Cohn SM, Proctor KG. Effects of arginine vasopressin during resuscitation from hemorrhagic hypotension after traumatic brain injury. Crit Care Med. 2006;34(2):433-8.

22. Rehberg S, Ermer C, Lange M et al. Role of selective V2 receptor antagonism in septic shock: a randomized, controlled, experimental study Crit.Care 2010,14:R200.

23. Dellinger RP, Levy MM, Rhodes A, Annane D, Gerlach H, Opal SM, Sevransky JE, Sprung CL, Douglas IS, Jaeschke R, Osborn TM, Nunnally ME, Townsend SR, Reinhart K, Kleinpell RM, Angus DC, Deutschman CS, Machado FR, Rubenfeld GD, Webb SA, Beale RJ, Vincent JL, Moreno R; Surviving Sepsis Campaign Guidelines Committee including the Pediatric Subgroup. Surviving sepsis campaign: international guidelines for management of severe sepsis and septic shock: 2012. Crit Care Med. 2013;41(2):580-637.

24. Delmas A, Leone M, Rousseau S, et al. Clinical review: vasopressin and terlipressin in septic shock patients. Crit Care 2005;9:212-22.

25. Johnson KB, Pearce FJ, Jeffreys N, McJames SW, Cluff M. Impact of vasopressin on hemodynamic and metabolic function in the decompensatory phase of hemorrhagic shock. J Cardiothorac Vasc Anesth. 2006;20(2):167-72.

26. Voelckel WG, Lurie KG, Lindner KH, et al: Vasopressin improves survival after cardiac arrest in hypovolemic shock. Anesth Analg 2000;91:627-634.

27. Haas, T., Voelckel, W.G., Wiedermann, F., Wenzel, V., Lindner, K.H. Successful resuscitation of a traumatic cardiac victim in hemorrhagic shock with vasopressin: a case report and brief review of the literature. J Trauma 2004;57:177–179.

28. Morales, D., Madigan, J., Cullinane S., Chen, J., Heath, M., Oz, M., Oliver, J.A., Landry, D.W. Reversal by vasopressin of intractable hypotension in the late phase of hemorrhagic shock. Circulation 1999;100: 226–229.

29. Wenzel V, Krismer AC, Arntz HR, et al. A comparison of vasopressin and epinephrine for out-of-hospital cardiopulmonary resuscitation. N Engl J Med 2004;350:105–113.

30. Stadlbauer KH, Wagner-Berger HG, Raedler C, et al. Vasopressin, but not fluid resuscitation, enhances survival in a liver trauma model with uncontrolled and otherwise lethal hemorrhagic shock in pigs. Anesthesiology. 2003;98:699–704.

31. Cavus E, Meybohm P, Doerges V, Hugo HH, Steinfath M, Nordstroem J, Scholz J, Bein B. Cerebral effects of three resuscitation protocols in uncontrolled haemorrhagic shock: A randomised controlled experimental study. Resuscitation. 2009;80(5):567-72.

32. Feinstein AJ, Cohn SM, King DR, Sanui M, Proctor KG. Early vasopressin improves short-term survival after pulmonary contusion. J Trauma. 2005 Oct;59(4):876-82.

33. Dunser MW, Mayr AJ, Tur A, et al. Ischemic skin lesions as a complication of continuous vasopressin infusion in catecholamine resistant vasodilatory shock: incidence and risk factors. Crit Care Med 2003;31:1394–1398.

34. Prengel AW, Lindner KH, Wenzel V, et al. Splanchnic and renal blood flow after cardiopulmonary resuscitation with epinephrine and vasopressin in pigs. Resuscitation 1998;38:19–24.

35. Szmydynger-Chodobska J, Chung I, Kozniewska E, et al. Increased expression of vasopressin v1a receptors after traumatic brain injury. J Neurotrauma 2004;21:1090–1102.

36. Biondi-Zoccai GGL, Agostoni P, Abbate A et al. A simple hint to improve Robinson and Dickersin’sw higly sensitive PubMed search strategy for controlled clinical trials. Int J Epidemiol 2005;34:224-25.

37. Zangrillo A, Biondi-Zoccai GG, Frati E, Covello RD, Cabrini L, Guarracino F, Ruggeri L, Bove T, Bignami E, Landoni G. Fenoldopam and acute renal failure in cardiac surgery: a meta-analysis of randomized placebo-controlled trials. J Cardiothorac Vasc Anesth. 2012 Jun;26(3):407-13.

38. Higgins JP, Thompson SG, Deeks JJ et al. Measuring inconsistency in meta-analysis. BMJ 2003;327:557-60.

39. Liu L, Tian K, Xue M, Zhu Y, Lan D, Peng X, Wu Y, Li T. Small Doses of Arginine Vasopressin in Combination With Norepinephrine "buy" Time for Definitive Treatment for Uncontrolled Hemorrhagic Shock in Rats. Shock. 2013 Oct. [Epub ahead of print]

40. Stadlbauer KH, Wagner-Berger HG, Krismer AC, Voelckel WG, Konigsrainer A, Lindner KH, Wenzel V. Vasopressin improves survival in a porcine model of abdominal vascular injury. Crit Care. 2007;11(4):R81.

41. Beloncle F, Meziani F, Lerolle M, Radermacher P, Asfar P. Does vasopressor therapy have an indication in hemorrhagic shock? Ann Intensive Care. 2013;22;3(1):13.

42. Oliver G, Schaefer E. On the physiological action of extract of pituitary body and certain other glandular organs. J Physiol 1895;18:277-279.

43. Landry DW, Levin HR, Gallant EM et al. Vasopressin pressor hypersensitivity in vasodilatory septic shock. Crit Care Med 1997;25:1279-82.

44. Russell JA, Walley KR, Singer J, Gordon AC, Hébert PC, Cooper DJ, Holmes CL, Mehta S, Granton JT, Storms MM, Cook DJ, Presneill JJ, Ayers D; VASST Investigators. Vasopressin versus norepinephrine infusion in patients with septic shock. N Engl J Med. 2008;358(9):877-87.

45. Lindner KH, Haak T, Keller A, Bothner U, Lurie KG. Release of endogenous vasopressors during and after cardiopulmonary resuscitation. Heart. 1996;75(2):145-50.

46. Lindner KH, Strohmenger HU, Ensinger H, Hetzel WD, Ahnefeld FW, Georgieff M. Stress hormone response during and after cardiopulmonary resuscitation. Anesthesiology. 1992;77(4):662-8.

47. Thiemermann C, Szabó C, Mitchell JA, Vane JR. Vascular hyporeactivity to vasoconstrictor agents and hemodynamic decompensation in hemorrhagic shock is mediated by nitric oxide. Proc Natl Acad Sci U S A. 1993;90(1):267-71.

48. Eltzschig HK, Carmeliet P. Hypoxia and inflammation. N Engl J Med. 2011;364(7):656-65.

49. Smaïl N, Descorps Declère A, Duranteau J, Vigué B, Samii K. Left ventricular function after severe trauma. Intensive Care Med. 1996;22(5):439-42.

50. Plurad DS, Talving P, Lam L, Inaba K, Green D, Demetriades D. Early vasopressor use in critical injury is associated with mortality independent from volume status. J Trauma. 2011;71(3):565-70.

51. Sharma RM, Setlur R. Vasopressin in hemorrhagic shock. Anesth Analg 2005;101:833-34.

52. Lienhart H, Wenzel V, Braun J et al. Vasopressin for therapy of persistent traumatic hemorrhagic shock: The VITRIS study. Anesthesist 2007;56:145-48.

53. Collier B, Dossett L, Mann M, Cotton B, Guillamondegui O, Diaz J, Fleming S, May A, Morris J. Vasopressin use is associated with death in acute trauma patients with shock. J Crit Care. 2010;25(1):173.e9-14.

54. Grmec S, Strnad M, Cander D, Mally S. A treatment protocol including vasopressin and hydroxyethyl starch solution is associated with increased rate of return of spontaneous circulation in blunt trauma patients with pulseless electrical activity. Int J Emerg Med. 2008;1(4):311-6.

55. Cavus E, Meybohm P, Doerges V, Hoecker J, Betz M, Hanss R, Steinfath M, Bein B. Effects of cerebral hypoperfusion on bispectral index: a randomised, controlled animal experiment during haemorrhagic shock. Resuscitation. 2010;81(9):1183-9.

56. Dudkiewicz M, Proctor KG. Tissue oxygenation during management of cerebral perfusion pressure with phenylephrine or vasopressin. Crit Care Med. 2008;36(9):2641-50.

57. Meybohm P, Cavus E, Dörges V, Weber B, Stadlbauer KH, Wenzel V, Scholz J, Steffen M, Bein B. Release of protein S100B in haemorrhagic shock: effects of small volume resuscitation combined with arginine vasopressin. Resuscitation 2008;76(3):449-56.

58. Lienhart HG, Lindner KH, Wenzel V. Developing alternative strategies for the treatment of traumatic haemorrhagic shock. Curr Opin Crit Care. 2008;14(3):247-53.

59. Dünser MW, Mayr AJ, Ulmer H, Ritsch N, Knotzer H, Pajk W, Luckner G, Mutz NJ, Hasibeder WR. The effects of vasopressin on systemic hemodynamics in catecholamine-resistant septic and postcardiotomy shock: a retrospective analysis. Anesth Analg. 2001;93(1):7-13.

60. Dunser MW, Wenzel V, Mayr AJ, Hasibeder WR. Management of vasodilatory shock: defining the role of arginine vasopressin. Drugs. 2003;63(3):237-56.

61 Sperry JL, Minei JP, Frankel HL, West MA, Harbrecht BG, Moore EE, Maier RV, Nirula R. Early use of vasopressors after injury: caution before constriction. J Trauma. 2008;64(1):9-14.

62. Beloncle F, Meziani F, Lerolle N, Radermacher P, Asfar P. Does vasopressor therapy have an indication in hemorrhagic shock? Ann Intensive Care. 2013;22;3(1):13.

63. Rajani RR, Ball CG, Feliciano DV, Vercruysse GA. Vasopressin in hemorrhagic shock: review article. Am Surg. 2009 Dec;75(12):1207-12.

64 Vesterinen HM, Sena ES, Egan KJ, Hirst TC, Churolov L, Currie GL, Antonic A, Howells DW, Macleod MR.Meta-analysis of data from animal studies: a practical guide. J Neurosci Methods. 2014 Jan 15;221:92-102. Epub 2013 Oct 4.
