# Supplementary material for: Vasopressin in Hemorrhagic Shock: A Systematic Review and Meta-Analysis of Randomized Animal Trials
Source: Biomed Res Int. 2014 Sep 1;2014:421291. doi: 10.1155/2014/421291 (PMC4165559; doi:10.1155/2014/421291)
Supplement: Supplementary file 1 — In the supplemental materials we confronted AVP/terlipressin with different comparators singularly: fluid resuscitation (fig. 6a), placebo (6b), other vasoconstrictive drugs(6C) and norepineprhine(6d). In all the analysis we conducted AVP/terlipressin was associated to a reduction of the death rate. We also did a meta-analysis on survival considering separately the studies conducted on rats (fig. 7a) and on pigs (fig.7b). In fig.8 we considered only the studies where hemorrhagic shock was due to a splancnic bleeding. We then did a meta-analysis excluding those trials with zero mortality (fig. 9) and selecting the studies that had mortality as the primary end-point. In table 3 are reported the dosages of AVP, terlipressin, vasopressors and the total amount of fluids included in the studies in the meta-analysis. In table 4 are reported the primary end-points and the setting of the included studies. [file 421291.f1.zip › supp/1038649.docx]

**Vasopressin in hemorrhagic shock: a systematic review and meta-analysis of randomized animal trials.**

Cossu AP1, Mura P1, De Giudici LM1, Pasin L2, Evangelista M3, Musu M1, Finco G1.

1 UOC Anesthesia and Intensive Care and, Pain Therapy Service, AOU Cagliari

Department of Medical Sciences “M. Aresu”, University of Cagliari, Italy.

2 Department of Anesthesia and Intensive Care, Vita-Salute San Raffaele University, Milan, Italy.

3 Department of Anesthesia and Intensive Care, Catholic University, Rome, Italy

Corresponding Author: Cossu Andrea Pasquale. [andreapcossu@yahoo.it](mailto:andreapcossu@yahoo.it)

Keywords: vasopressin, terlipressin, haemorrhagic shock, hypovolemic shock, vasopressors.

**Supplemental Material**

**
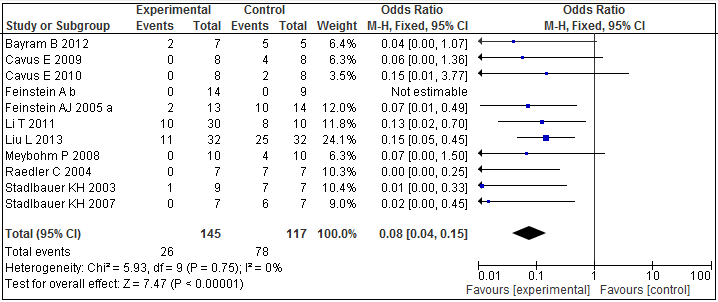
**

**Fig 6a: AVP or terlipressin vs fluid resuscitation**

**
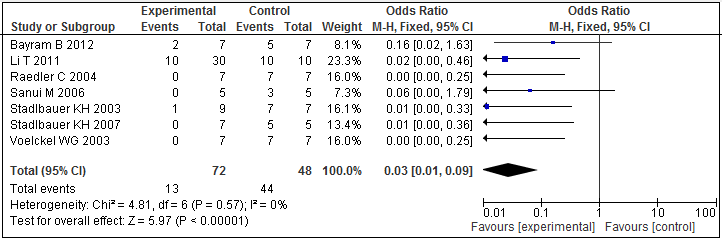
**

**Fig 6b: AVP or terlipressin vs placebo**

**
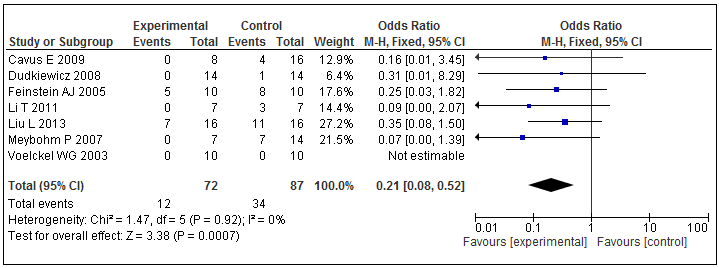
**

**Fig 6c: AVP vs other vasoconstrictive drugs**

**
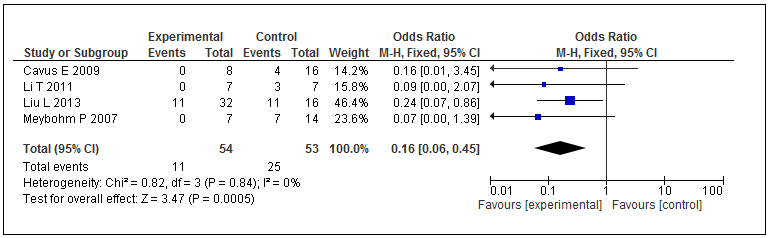
Fig 6d: AVP vs NE
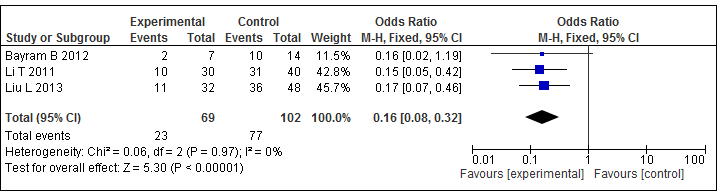
Fig. 7a: AVP or terlipressin vs all other strategies in rats
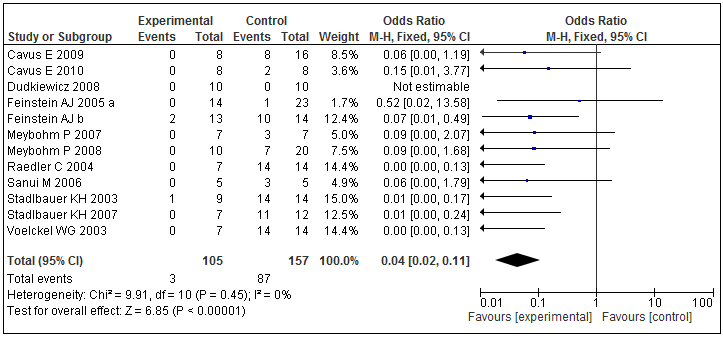
Fig. 7b: AVP vs all other strategies in pigs.
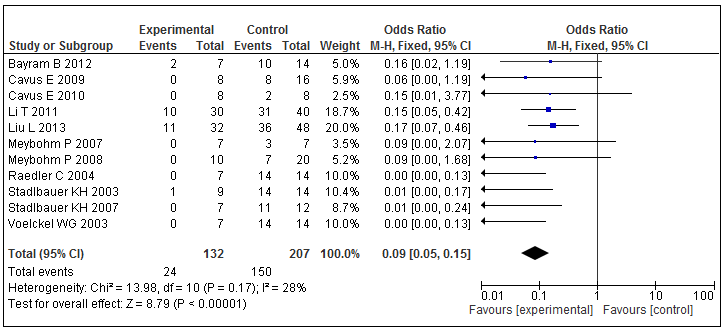
Fig. 8: Meta-analysis performed considering only studies were hemorrhagic shock was due to bleeding occurring in the splanchnic area.**

**
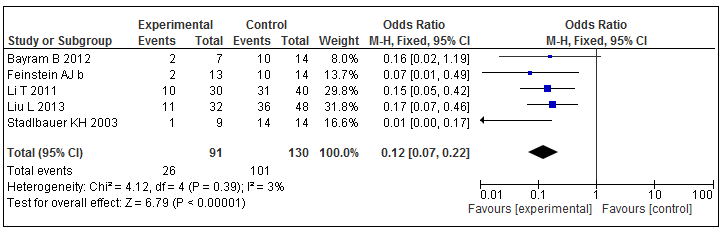
**

**Fig. 9: meta-analysis performed excluding studies with zero mortality rate.**

**
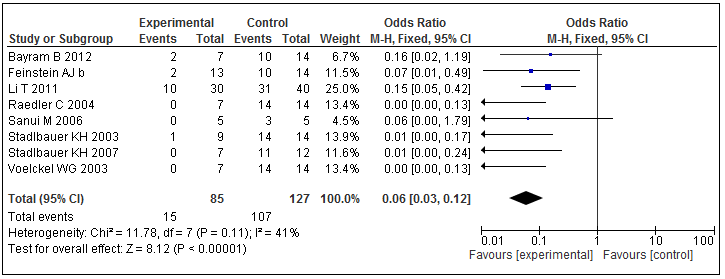
**

**Fig, 10:meta-analysis performed considering only studies that used mortality as the primary endpoint**

| **Trial** | **AVP or terlipressin dosages** | **Fluid administration in AVP group** | **Vasopressors dosages**  **In control groups** | **Fluid administration in vasopressor groups** | **Fluid administration in fluid resuscitation groups** | **AVP + VASOPRESSORS** |
| --- | --- | --- | --- | --- | --- | --- |
| **Bayram B [3]** | Terlipressin: 50 μg/kg | LR 2 mL/kg per min  (PAM target 40 mmHg) | - | - | Bolus of LR (4 mL/kg) + 2 mL/kg/min (PAM target 40 mmHg) | NO |
| **Cavus E [31]** | AVP: bolus of 0.2U/kg  + infusion 2U/kg/h | HSS 4mL/kg  over 2min | - | - | LR (40mL/kg) +HES 130/0.4 (20mL/kg) over 30min | NO |
| **Cavus E [55]** | AVP: bolus 0.2U/kg + infusion 0.04U/kg/min | HSS (4mL/kg) over 2min | NE (bolus 20 ɣg/kg + 1 ɣg/kg/min infusion) | HSS (4mL/kg) over 2min | 6% HES 130/0.4 (20 mL/kg) + LR (40 mL/kg) over 30 min; | NO |
| **Dudkiewicz M [56]** | AVP titrated to maintain a CPP > 70 mm Hg and CVP > 12 mm Hg | NS 1 L + unlimited NS to SAP>100 plus 250 mg/kg mannitol | NE titrated to maintain a CPP > 70 mm Hg and CVP > 12 mm Hg | NS 1 L + unlimited NS to SAP>100 plus 250 mg/kg mannitol | - | NO |
| **Feinstein AJ [8]** | AVP: 0.1 U/kg  + bolus to MAP > 60 mmHg | NS titrated to MAP > 60 mmHg (only 1 group) | PE: 0.05 mg/kg  + bolus to MAP > 60 mmHg | NS titrated to MAP > 60 mmHg (only 1 group) | NS titrated to MAP > 60 mmHg | NO |
| **Feinstein AJ [32]** | AVP 0.1 U/kg + continuous AVP (0.4U/kg/hr) | NS bolus 10 mL/kg + NS was infused to MAP>70mmHg | - | - | NS bolus 10 mL/kg + NS was infused to MAP > 70 mmHg | NO |
| **Li T [11]** | AVP 0.1 U/kg | Two volume of LR | NE 1 mg/kg | Two volumes of LR | Two volumes of LR solution; One volume of blood + one volume of LR | AVP 0.1 U/kg + NE 1 mg/kg + LR 2 volumes or blood 1 volume + LR 1 volume |
| **Liu L [39]** | AVP 0.04 or 0.4 U/kg | LR (17.5 mL/kg or 8.75mL/kg or 1 mL/kg) | NE 3 µg/kg | LR (17.5 mL/kg or 8.75mL/kg or 1 mL/kg) | LR (17.5 mL/kg or 8.75mL/kg or 1 mL/kg) | AVP 0.04 or 0.4 U/kg + NE 3 µg/kg + LR (17.5 mL/kg or 8.75mL/kg or 1 mL/kg) |
| **Meybohm P [13]** | AVP bolus 10 U +  2 U/kg/hr | HHS 4 mL/kg  during 2 minutes | NE 1000 ɣg + infusion of 60 ɣg/kg/hr | HHS 4 mL/kg  during 2 minutes | - | NO |
| **Meybohm P [57]** | AVP bolus 0.2Ukg + 2 UI/kg/h | HHS (4 mL/kg over 2min) + LR 10 ml/kg/h and HES 10 ml/kg H | - | - | LR (40 mL/kg) and HES 130/0.4 (20mLkg) + LR 10 ml/kg/h and HES 10 ml/kg H | NO |
| **Raedler C [10]** | AVP 0.4 U/kg bolus + 0.04 ml/kg/min | HES 1000 mL and  LR 1000 mL  (after 30 min) | - | - | LR 1000 Ml + HES 1000 mL (up to 8 ml/kg/min) + HES 1000 mL and  LR 1000 mL after 30 min | NO |
| **Sanui M [21]** | AVP 0.2 U/kg bolus + 0.1 U/kg/min | NS 10 mL/kg + crystalloids MAP target > 60 mm Hg | - | - | NS 10 mL/kg + crystalloids MAP target > 60 mm Hg | NO |
| **Stadlbauer KH [30]** | AVP 0.4 U/kg + 0.08 U/kg/min | Whole blood (~40 ml/kg) LR 25 ml/kg and 25 ml/kg 3% gelatin solution | - | - | LR 25 ml/kg and 25 ml/kg 3% gelatin solution | NO |
| **Stadlbauer KH [40]** | AVP 0.4 U/kg | LR 25 mL/kg + 25 mL/kg 3% gelatin (after 30 min) | - | . | LR 25 mL/kg | NO |
| **Voelckel WG [1]** | AVP 0.8 U/kg | 3 mL/kg/h | PE 200 ɣg/kg | 3 mL/kg/h | - | NO |

**Tab. 3: dosages of AVP/terlipressin, vasopressors and fluid resuscitation in studies included in meta-analysis. LR: Lactate Ringer; HES: hydroxyethylstarch; HSS: hypertonic-saline-starch solution; CPP: cerebral perfusion pressure; CVP: central venous pressure; PE: phenylephrine; NS, normal saline; HHS: HyperHAES**

| STUDY  (First Author and Year of pubblication) | PRIMARY ENDPOINT OF THE STUDY | SETTING |
| --- | --- | --- |
| **Bayram B. 2012** | Increase in MAP  Survival rates | Liver Trauma |
| **Cavus E 2010** | Changes of bis-pectral index (BIS)  Changes of cerebral perfusion | Liver Trauma |
| **Cavus E 2009** | Haemodynamic and cerebral variables | Liver Trauma |
| **Dudkiewicz M 2008** | Maintain tissue oxygenation during cerebral perfusion pressure management | Blunt trauma to the head and bilateral chests |
| **Feinstein AJ 2005** | ICP  Physiologic resuscitation parameters | Percussion traumatic brain Injury followed by hemorrhage |
| **Feinstein AJ 2005** | Mortality  Fluid requirement  Pulmonary function | Severe chest trauma |
| **Li T 2011** | 12-h animal survival rate  Tissue blood flow  Mitochondrial function of liver and kidney | Splenic parenchyma and one of  splenic artery transection |
| **Liu L 2013** | Maintain and stabilize hemodynamic parameters | Transection of the splenic parenchyma, the splenic artery and vein |
| **Meybohm P 2007** | Cerebral perfusion pressure (CPP) and  brain metabolism | Liver bleeding |
| **Meybohm P 2008** | Cerebral perfusion pressure (CPP) and protein S100B | Liver trauma |
| **Raedler C 2004** | Hemodynamic variables  Short-term survival | Liver trauma |
| **Sanui M 2006** | Intracranial pressure (ICP)  Survival | Traumatic brain injury |
| **Stadlbauer KH 2003** | Hemodynamic variables  Survival | Liver trauma |
| **Stadlbauer KH 2007** | Hemodynamic variables  Survival | Abdominal vascular injury |
| **Voelckel WG 2003** | Hemodynamic variables  Regional blood flow  Short-term survival | Liver trauma |

**Tab. 4: Primary endpoint of animal studies**
