# Supplementary material for: Vasopressin in Hemorrhagic Shock: A Systematic Review and Meta-Analysis of Randomized Animal Trials
Source: Biomed Res Int. 2014 Sep 1;2014:421291. doi: 10.1155/2014/421291 (PMC4165559; doi:10.1155/2014/421291)
Supplement: Supplementary file 1 — In the supplemental materials we confronted AVP/terlipressin with different comparators singularly: fluid resuscitation (fig. 6a), placebo (6b), other vasoconstrictive drugs(6C) and norepineprhine(6d). In all the analysis we conducted AVP/terlipressin was associated to a reduction of the death rate. We also did a meta-analysis on survival considering separately the studies conducted on rats (fig. 7a) and on pigs (fig.7b). In fig.8 we considered only the studies where hemorrhagic shock was due to a splancnic bleeding. We then did a meta-analysis excluding those trials with zero mortality (fig. 9) and selecting the studies that had mortality as the primary end-point. In table 3 are reported the dosages of AVP, terlipressin, vasopressors and the total amount of fluids included in the studies in the meta-analysis. In table 4 are reported the primary end-points and the setting of the included studies. [file 421291.f1.zip › supp/1038655.docx]

| **1st Author** | **Journal** | **Year** | **N° AVP**(V) **or Terlipressin**(T) | **N° Control** | **Control** | **Animal** |
| --- | --- | --- | --- | --- | --- | --- |
| **Bayram B [3]** | Am J Emerg Med | 2012 | 7 (T) | 14 | Placebo (7); Ringer Lactate (7) | Rats |
| **Cavus E [31]** | Resuscitation | 2010 | 8 (V) | 8 | Fluid resuscitation (8) | Pigs |
| **Cavus E [55]** | Resuscitation | 2009 | 8 (V) | 16 | Fluid resuscitation (8);  Noradrenaline + HS (8) | Pigs |
| **Dudkiewicz M [56]** | Crit Care Med | 2008 | 10 (V) | 10 | Phenylephrine (10) | Pigs |
| **Feinstein AJ [8]** | J Am Coll Surg | 2005 | 14 (V) | 23 | Crystalloid (9); Phenylephrine(5);  Crystalloid + phenylephrine(9) | Pigs |
| **Feinstein AJ [32]** | J Trauma | 2005 | 8 (V) | 9 | NS (9) | Pigs |
| **Li T [11]** | J Surg Res | 2011 | 30 (V) | 40 | Placebo (10); Ringer Lactate (10); Whole blood (10);NE (10) | Rats |
| **Liu L [39]** | Shock | 2013 | 32 (V) | 48 | Hypotensive resuscitation (16);  Ringer Lactate (16); NE (16) | Rats |
| **Meybohm P [13]** | J Trauma | 2007 | 7 (V) | 7 | HHS + NE (7) | Pigs |
| **Meybohm P [57]** | Resuscitation | 2008 | 10 (V) | 20 | Fluid (10); HHS+NS (10) | Pigs |
| **Raedler C [10]** | Anesth Analg | 2004 | 7 (V) | 14 | Saline placebo (7);  Fluid resuscitation (7) | Pigs |
| **Sanui M [21]** | Crit Care Med | 2006 | 5 (V) | 5 | Placebo (5) | Pigs |
| **Stadlbauer KH [30]** | Anesthesiology | 2003 | 9 (V) | 14 | Saline placebo (7);  Fluid resuscitation (7) | Pigs |
| **Stadlbauer KH [40]** | Crit Care | 2007 | 7 (V) | 12 | Saline placebo (5);  Fluid resuscitation (7) | Pigs |
| **Voelckel WG [1]** | Crit Care Med | 2003 | 7 (V) | 14 | Epinephrine (7);  Saline placebo (7) | Pigs |

**Table 1: studies included in the meta-analysis**
