# Supplementary material for: Vasopressin in Hemorrhagic Shock: A Systematic Review and Meta-Analysis of Randomized Animal Trials
Source: Biomed Res Int. 2014 Sep 1;2014:421291. doi: 10.1155/2014/421291 (PMC4165559; doi:10.1155/2014/421291)
Supplement: Supplementary file 1 — In the supplemental materials we confronted AVP/terlipressin with different comparators singularly: fluid resuscitation (fig. 6a), placebo (6b), other vasoconstrictive drugs(6C) and norepineprhine(6d). In all the analysis we conducted AVP/terlipressin was associated to a reduction of the death rate. We also did a meta-analysis on survival considering separately the studies conducted on rats (fig. 7a) and on pigs (fig.7b). In fig.8 we considered only the studies where hemorrhagic shock was due to a splancnic bleeding. We then did a meta-analysis excluding those trials with zero mortality (fig. 9) and selecting the studies that had mortality as the primary end-point. In table 3 are reported the dosages of AVP, terlipressin, vasopressors and the total amount of fluids included in the studies in the meta-analysis. In table 4 are reported the primary end-points and the setting of the included studies. [file 421291.f1.zip › supp/1038656.docx]

| **Outcome** | **Number of included trials** | **AVP/terlipressin**  **animals** | **Control**  **animals** | **OR** | **95% CI** | **P for effect** | **P for heterogeneity** | **I2 (%)** |
| --- | --- | --- | --- | --- | --- | --- | --- | --- |
| **Overall trials** | 15 | 174 | 259 | 0.09 | 0.05-0.15 | < 0.001 | 0.30 | 14 |
| **Mortality** |  | 15% | 63% |  |  |  |  |  |
| **Placebo as comparator drug** | 7 | 72 | 48 | 0.03 | 0.01-0.09 | < 0.001 | 0.57 | 0 |
| **Mortality** |  | 18% | 92% |  |  |  |  |  |
| **Fluid resuscitation as comparator drug** | 11 | 114 | 117 | 0.08 | 0.04-0.15 | < 0.001 | 0.75 | 0 |
| **Mortality** |  | 18% | 67% |  |  |  |  |  |
| **Vasopressors (NE or epinephrine) as comparator drug** | 7 | 88 | 87 | 0.18 | 0.08-0.44 | < 0.001 | 0.96 | 0 |
| **Mortality** |  | 18% | 39% |  |  |  |  |  |
| **NE as comparator**  **drug** | 4 | 54 | 53 | 0.16 | 0.06-0.45 | < 0.001 | 0.97 | 0 |
| **Mortality** |  | 20% | 47% |  |  |  |  |  |
| **SENSITIVITY ANALYSIS** (including only low risk of bias studies) | 10 | 134 | 195 | 0.13 | 0.08-0.24 | < 0.001 | 0.99 | 0 |
| **Mortality** |  | 18% | 57% |  |  |  |  |  |

**Table 2: Results for mortality**
