# Supplementary material for: Vasopressin in Hemorrhagic Shock: A Systematic Review and Meta-Analysis of Randomized Animal Trials
Source: Biomed Res Int. 2014 Sep 1;2014:421291. doi: 10.1155/2014/421291 (PMC4165559; doi:10.1155/2014/421291)
Supplement: Supplementary file 1 — In the supplemental materials we confronted AVP/terlipressin with different comparators singularly: fluid resuscitation (fig. 6a), placebo (6b), other vasoconstrictive drugs(6C) and norepineprhine(6d). In all the analysis we conducted AVP/terlipressin was associated to a reduction of the death rate. We also did a meta-analysis on survival considering separately the studies conducted on rats (fig. 7a) and on pigs (fig.7b). In fig.8 we considered only the studies where hemorrhagic shock was due to a splancnic bleeding. We then did a meta-analysis excluding those trials with zero mortality (fig. 9) and selecting the studies that had mortality as the primary end-point. In table 3 are reported the dosages of AVP, terlipressin, vasopressors and the total amount of fluids included in the studies in the meta-analysis. In table 4 are reported the primary end-points and the setting of the included studies. [file 421291.f1.zip › supp/1045077.pptx]

## Slide 1
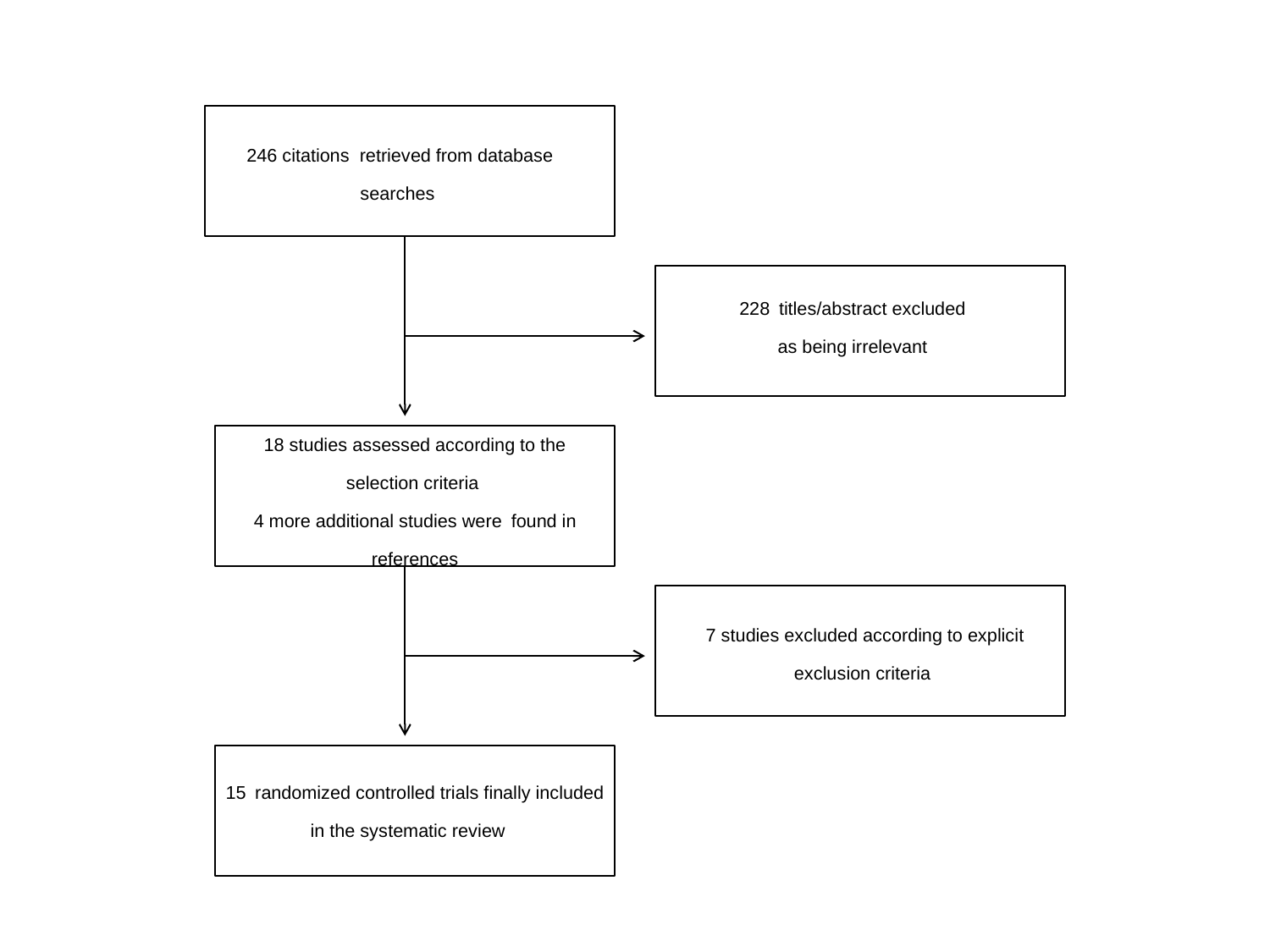

246 citations retrieved from database searches
228 titles/abstract excluded
as being irrelevant
18 studies assessed according to the selection criteria
4 more additional studies were found in references
7 studies excluded according to explicit exclusion criteria
15 randomized controlled trials finally included in the systematic review
