# Supplementary material for: Vasopressin in Hemorrhagic Shock: A Systematic Review and Meta-Analysis of Randomized Animal Trials
Source: Biomed Res Int. 2014 Sep 1;2014:421291. doi: 10.1155/2014/421291 (PMC4165559; doi:10.1155/2014/421291)
Supplement: Supplementary file 1 — In the supplemental materials we confronted AVP/terlipressin with different comparators singularly: fluid resuscitation (fig. 6a), placebo (6b), other vasoconstrictive drugs(6C) and norepineprhine(6d). In all the analysis we conducted AVP/terlipressin was associated to a reduction of the death rate. We also did a meta-analysis on survival considering separately the studies conducted on rats (fig. 7a) and on pigs (fig.7b). In fig.8 we considered only the studies where hemorrhagic shock was due to a splancnic bleeding. We then did a meta-analysis excluding those trials with zero mortality (fig. 9) and selecting the studies that had mortality as the primary end-point. In table 3 are reported the dosages of AVP, terlipressin, vasopressors and the total amount of fluids included in the studies in the meta-analysis. In table 4 are reported the primary end-points and the setting of the included studies. [file 421291.f1.zip › supp/mat.421291.v2.pdf]

Vasopressin in hemorrhagic shock: a systematic review and meta-analysis of randomized animal trials.

Cossu AP1, Mura P1, De Giudici LM1, Pasin L2, Evangelista M3, Musu M1, Finco G1.

[1] UOC Anesthesia and Intensive Care and, Pain Therapy Service, AOU Cagliari Department of Medical Sciences “M. Aresu”, University of Cagliari, Italy.

[2] Department of Anesthesia and Intensive Care, Vita-Salute San Raffaele University, Milan, Italy.

[3] Department of Anesthesia and Intensive Care, Catholic University, Rome, Italy

Corresponding Author: Cossu Andrea Pasquale. andreapcossu@yahoo.it

Keywords: vasopressin, terlipressin, haemorrhagic shock, hypovolemic shock, vasopressors.

Supplemental Material

Study or Subgroup	Experimental Events	Experimental Total	Control Events	Control Total	Weight	Odds Ratio M-H, Fixed, 95% CI
Bayram B 2012	2	7	5	5	6.4%	0.04 [0.00, 1.07]
Cavus E 2009	0	8	4	8	6.3%	0.06 [0.00, 1.36]
Cavus E 2010	0	8	2	8	3.5%	0.15 [0.01, 3.77]
Feinstein A b	0	14	0	9		Not estimable
Feinstein AJ 2005 a	2	13	10	14	12.0%	0.07 [0.01, 0.49]
Li T 2011	10	30	8	10	11.8%	0.13 [0.02, 0.70]
Liu L 2013	11	32	25	32	24.1%	0.15 [0.05, 0.45]
Meybohm P 2008	0	10	4	10	6.3%	0.07 [0.00, 1.50]
Raedler C 2004	0	7	7	7	10.3%	0.00 [0.00, 0.25]
Stadlbauer KH 2003	1	9	7	7	10.4%	0.01 [0.00, 0.33]
Stadlbauer KH 2007	0	7	6	7	9.0%	0.02 [0.00, 0.45]
**Total (95% CI)**		**145**		**117**	**100.0%**	**0.08 [0.04, 0.15]**
Total events	26		78			

Heterogeneity: Chi² = 5.93, df = 9 (P = 0.75); I² = 0%

Test for overall effect: Z = 7.47 (P < 0.00001)

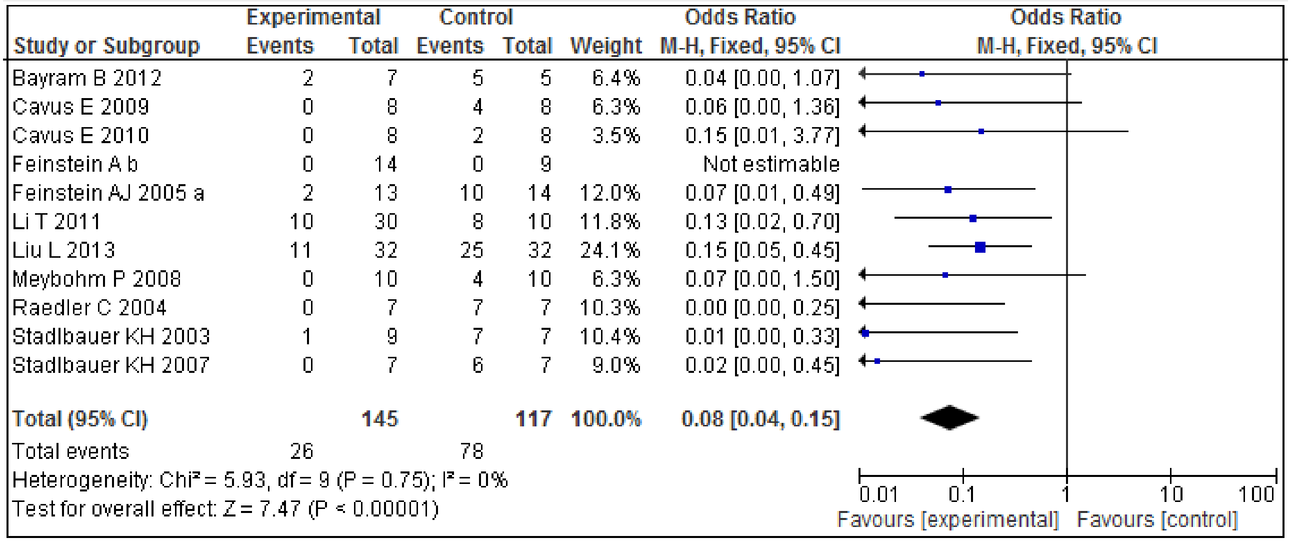

Fig 6a: AVP or terlipressin vs fluid resuscitation

Study or Subgroup	Experimental Events	Experimental Total	Control Events	Control Total	Weight	Odds Ratio M-H, Fixed, 95% CI
Bayram B 2012	2	7	5	7	8.1%	0.16 [0.02, 1.63]
Li T 2011	10	30	10	10	23.3%	0.02 [0.00, 0.46]
Raedler C 2004	0	7	7	7	16.0%	0.00 [0.00, 0.25]
Sanui M 2006	0	5	3	5	7.3%	0.06 [0.00, 1.79]
Stadlbauer KH 2003	1	9	7	7	16.1%	0.01 [0.00, 0.33]
Stadlbauer KH 2007	0	7	5	5	13.4%	0.01 [0.00, 0.36]
Voelckel WG 2003	0	7	7	7	16.0%	0.00 [0.00, 0.25]
**Total (95% CI)**		**72**		**48**	**100.0%**	**0.03 [0.01, 0.09]**
Total events	13		44			

Heterogeneity: Chi² = 4.81, df = 6 (P = 0.57); I² = 0%
Test for overall effect: Z = 5.97 (P < 0.00001)

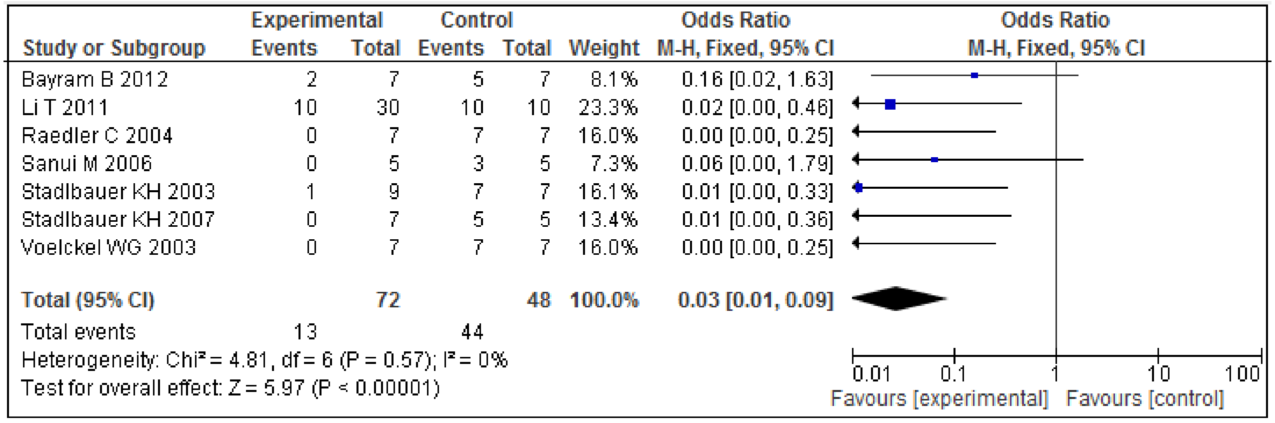

Fig 6b: AVP or terlipressin vs placebo

Study or Subgroup	Experimental Events	Experimental Total	Control Events	Control Total	Weight	Odds Ratio M-H, Fixed, 95% CI
Cavus E 2009	0	8	4	16	12.9%	0.16 [0.01, 3.45]
Dudkiewicz 2008	0	14	1	14	6.4%	0.31 [0.01, 8.29]
Feinstein AJ 2005	5	10	8	10	17.6%	0.25 [0.03, 1.82]
Li T 2011	0	7	3	7	14.4%	0.09 [0.00, 2.07]
Liu L 2013	7	16	11	16	27.2%	0.35 [0.08, 1.50]
Meybohm P 2007	0	7	7	14	21.5%	0.07 [0.00, 1.39]
Voelckel WG 2003	0	10	0	10		Not estimable
**Total (95% CI)**		**72**		**87**	**100.0%**	**0.21 [0.08, 0.52]**
Total events	12		34			

Heterogeneity: Chi² = 1.47, df = 5 (P = 0.92); I² = 0%
Test for overall effect: Z = 3.38 (P = 0.0007)

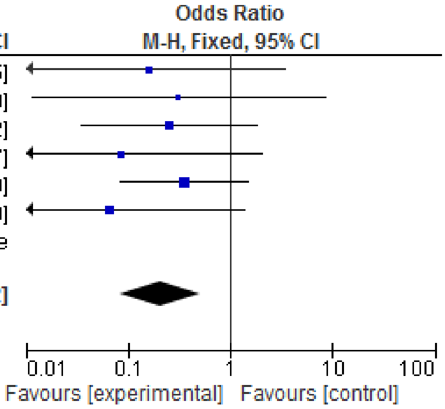

Fig 6c: AVP vs other vasoconstrictive drugs

Study or Subgroup	Experimental Events	Experimental Total	Control Events	Control Total	Weight	Odds Ratio M-H, Fixed, 95% CI
Cavus E 2009	0	8	4	16	14.2%	0.16 [0.01, 3.45]
Li T 2011	0	7	3	7	15.8%	0.09 [0.00, 2.07]
Liu L 2013	11	32	11	16	46.4%	0.24 [0.07, 0.86]
Meybohm P 2007	0	7	7	14	23.6%	0.07 [0.00, 1.39]
**Total (95% CI)**		**54**		**53**	**100.0%**	**0.16 [0.06, 0.45]**
Total events	11		25			

Heterogeneity: Chi² = 0.82, df = 3 (P = 0.84); I² = 0%
Test for overall effect: Z = 3.47 (P = 0.0005)

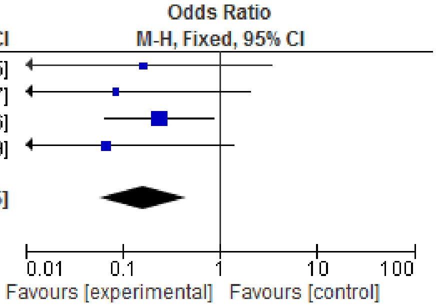

Fig 6d: AVP vs NE

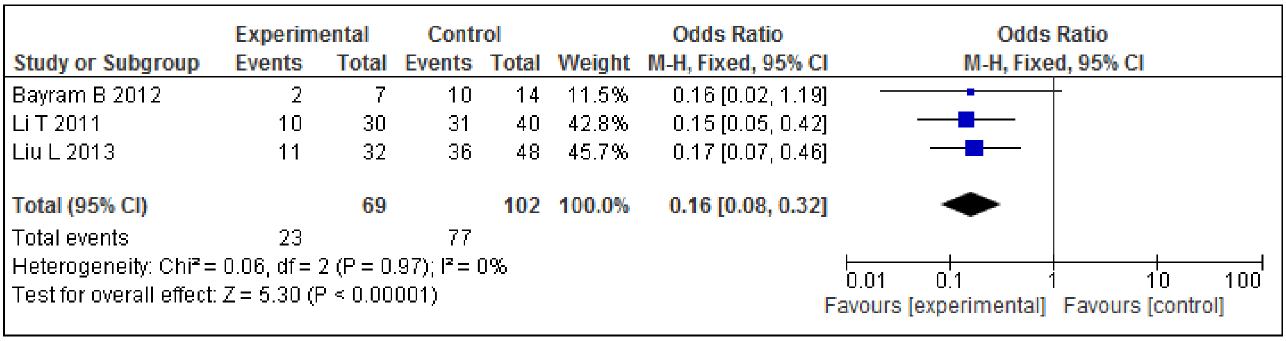

Fig. 7a: AVP or terlipressin vs all other strategies in rats

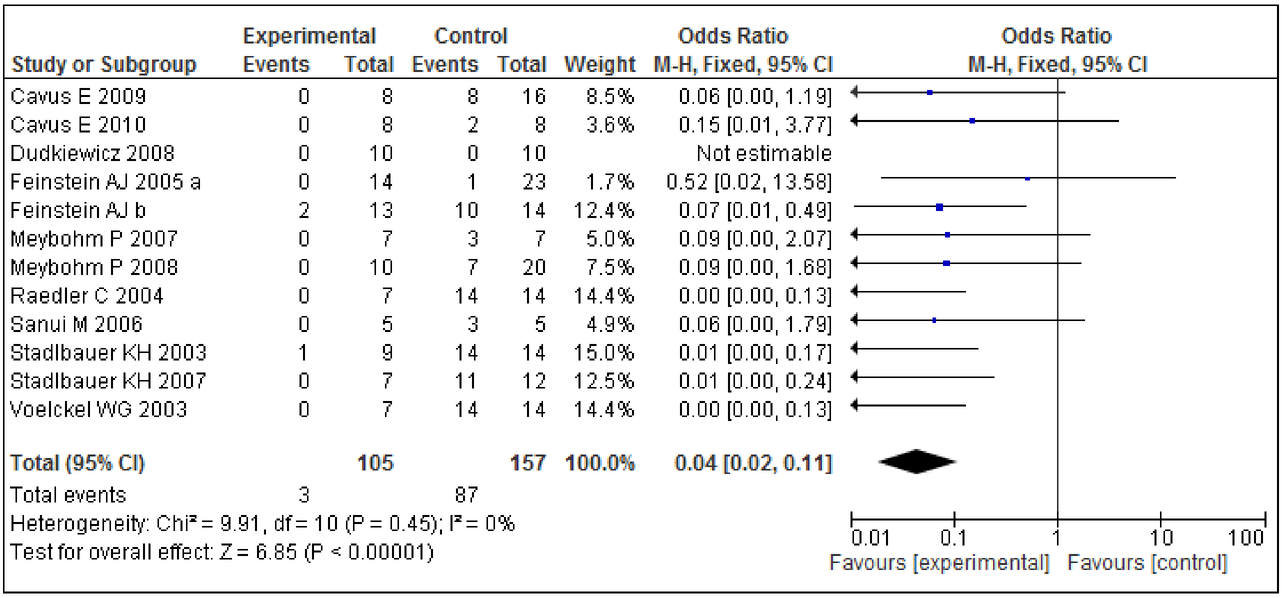

Fig. 7b: AVP vs all other strategies in pigs.

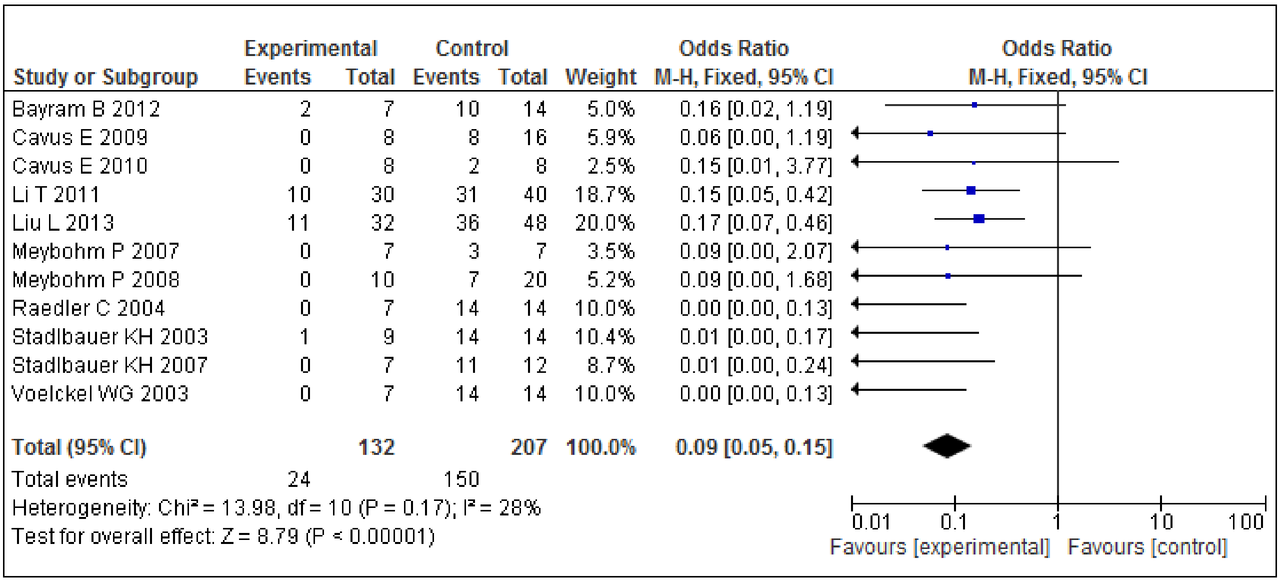

Fig. 8: Meta-analysis performed considering only studies were hemorrhagic shock was due to bleeding occurring in the splanchnic area.

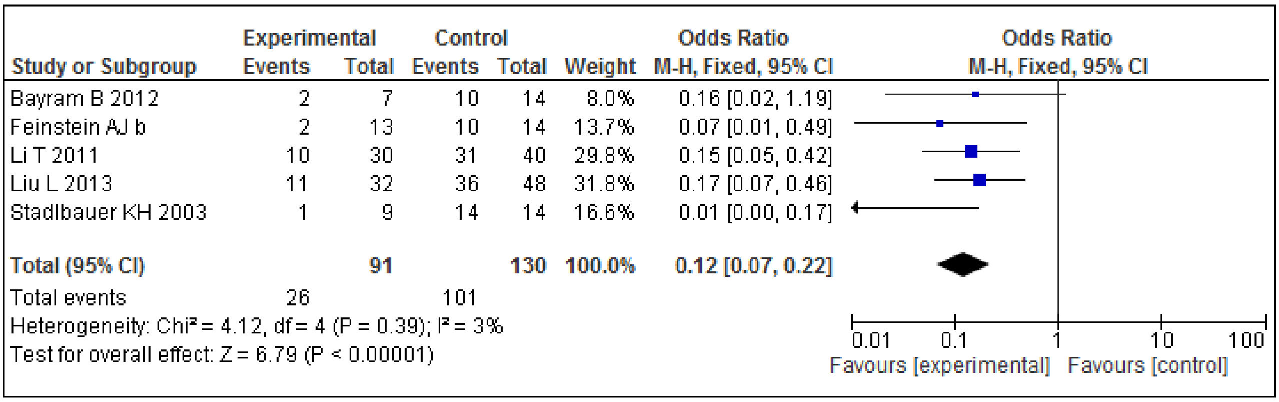

Fig. 9: meta-analysis performed excluding studies with zero mortality rate.

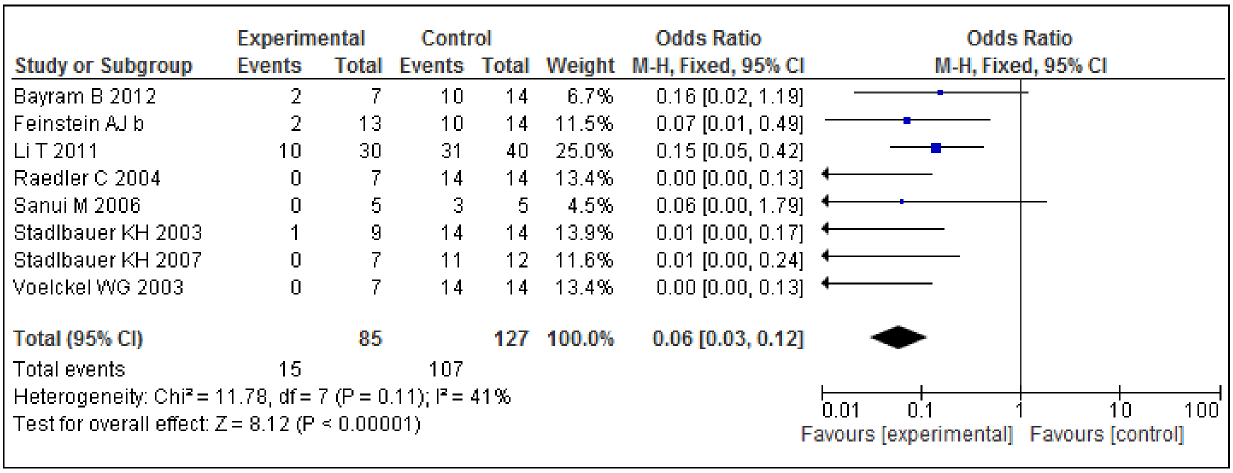

Fig, 10:meta-analysis performed considering only studies that used mortality as the primary endpoint

Trial	AVP or terlipressin dosages	Fluid administration in AVP group	Vasopressors dosages In control groups	Fluid administration in vasopressor groups	Fluid administration in fluid resuscitation groups	AVP + VASOPRESSORS
**Bayram B [3]**	Terlipressin: 50 µg/kg	LR 2 mL/kg per min (PAM target 40 mmHg)	-	-	Bolus of LR (4 mL/kg) + 2 mL/kg/min (PAM target 40 mmHg)	NO
**Cavus E [31]**	AVP: bolus of 0.2U/kg + infusion 2U/kg/h	HSS 4mL/kg over 2min	-	-	LR (40mL/kg) +HES 130/0.4 (20mL/kg) over 30min	NO
**Cavus E [55]**	AVP: bolus 0.2U/kg + infusion 0.04U/kg/min	HSS (4mL/kg) over 2min	NE (bolus 20 ɣg/kg + 1 ɣg/kg/min infusion)	HSS (4mL/kg) over 2min	6% HES 130/0.4 (20 mL/kg) + LR (40 mL/kg) over 30 min;	NO
**Dudkiewicz M [56]**	AVP titrated to maintain a CPP > 70 mm Hg and CVP > 12 mm Hg	NS 1 L + unlimited NS to SAP>100 plus 250 mg/kg mannitol	NE titrated to maintain a CPP > 70 mm Hg and CVP > 12 mm Hg	NS 1 L + unlimited NS to SAP>100 plus 250 mg/kg mannitol	-	NO
**Feinstein AJ**	AVP: 0.1 U/kg	NS titrated to MAP > 60	PE: 0.05 mg/kg + bolus to MAP	NS titrated to MAP > 60	NS titrated to	NO

**[8]**	+ bolus to MAP > 60 mmHg	mmHg (only 1 group)	> 60 mmHg	mmHg (only 1 group)	MAP > 60 mmHg	
**Feinstein AJ [32]**	AVP 0.1 U/kg + continuous AVP (0.4U/kg/hr)	NS bolus 10 mL/kg + NS was infused to MAP>70mmHg	-	-	NS bolus 10 mL/kg + NS was infused to MAP > 70 mmHg	NO
**Li T [11]**	AVP 0.1 U/kg	Two volume of LR	NE 1 mg/kg	Two volumes of LR	Two volumes of LR solution; One volume of blood + one volume of LR	AVP 0.1 U/kg + NE 1 mg/kg + LR 2 volumes or blood 1 volume + LR 1 volume
**Liu L [39]**	AVP 0.04 or 0.4 U/kg	LR (17.5 mL/kg or 8.75mL/kg or 1 mL/kg)	NE 3 µg/kg	LR (17.5 mL/kg or 8.75mL/kg or 1 mL/kg)	LR (17.5 mL/kg or 8.75mL/kg or 1 mL/kg)	AVP 0.04 or 0.4 U/kg + NE 3 µg/kg + LR (17.5 mL/kg or 8.75mL/kg or 1 mL/kg)

**Meybohm P [13]**	AVP bolus 10 U + 2 U/kg/hr	HHS 4 mL/kg during 2 minutes	NE 1000 ɣg + infusion of 60 ɣg/kg/hr	HHS 4 mL/kg during 2 minutes	-	NO
**Meybohm P [57]**	AVP bolus 0.2Ukg + 2 UI/kg/h	HHS (4 mL/kg over 2min) + LR 10 ml/kg/h and HES 10 ml/kg H	-	-	LR (40 mL/kg) and HES 130/0.4 (20mLkg) + LR 10 ml/kg/h and HES 10 ml/kg H	NO
**Raedler C [10]**	AVP 0.4 U/kg bolus + 0.04 ml/kg/min	HES 1000 mL and LR 1000 mL (after 30 min)	-	-	LR 1000 Ml + HES 1000 mL (up to 8 ml/kg/min) + HES 1000 mL and LR 1000 mL after 30 min	NO
**Sanui M [21]**	AVP 0.2 U/kg bolus + 0.1 U/kg/min	NS 10 mL/kg + crystalloids MAP target > 60 mm Hg	-	-	NS 10 mL/kg + crystalloids MAP target > 60 mm Hg	NO
**Stadlbauer KH [30]**	AVP 0.4 U/kg + 0.08 U/kg/min	Whole blood (~40 ml/kg) LR 25 ml/kg and 25 ml/kg 3% gelatin solution	-	-	LR 25 ml/kg and 25 ml/kg 3% gelatin solution	NO

**Stadlbauer KH [40]**	AVP 0.4 U/kg	LR 25 mL/kg + 25 mL/kg 3% gelatin (after 30 min)	-	.	LR 25 mL/kg	NO
**Voelckel WG [1]**	AVP 0.8 U/kg	3 mL/kg/h	PE 200 ɣg/kg	3 mL/kg/h	-	NO

Tab. 3: dosages of AVP/terlipressin, vasopressors and fluid resuscitation in studies included in meta-analysis. LR: Lactate Ringer; HES: hydroxyethylstarch; HSS: hypertonic-saline-starch solution; CPP: cerebral perfusion pressure; CVP: central venous pressure; PE: phenylephrine; NS, normal saline; HHS: HyperHAES

STUDY (First Author and Year of pubblication)	PRIMARY ENDPOINT OF THE STUDY	SETTING
**Bayram B. 2012**	Increase in MAP<br>Survival rates	Liver Trauma
**Cavus E 2010**	Changes of bis-pectral index (BIS)<br>Changes of cerebral perfusion	Liver Trauma
**Cavus E 2009**	Haemodynamic and cerebral variables	Liver Trauma
**Dudkiewicz M 2008**	Maintain tissue oxygenation during cerebral perfusion pressure management	Blunt trauma to the head and bilateral chests
**Feinstein AJ 2005**	ICP<br>Physiologic resuscitation parameters	Percussion traumatic brain Injury followed by hemorrhage
**Feinstein AJ 2005**	Mortality<br>Fluid requirement<br>Pulmonary function	Severe chest trauma
**Li T 2011**	12-h animal survival rate<br>Tissue blood flow<br>Mitochondrial function of liver and kidney	Splenic parenchyma and one of splenic artery transection
**Liu L 2013**	Maintain and stabilize hemodynamic parameters	Transection of the splenic parenchyma, the splenic artery and vein
**Meybohm P 2007**	Cerebral perfusion pressure (CPP) and brain metabolism	Liver bleeding
**Meybohm P 2008**	Cerebral perfusion pressure (CPP) and protein S100B	Liver trauma
**Raedler C 2004**	Hemodynamic variables<br>Short-term survival	Liver trauma
**Sanui M 2006**	Intracranial pressure (ICP)<br>Survival	Traumatic brain injury

**Stadlbauer KH 2003**	Hemodynamic variables<br>Survival	Liver trauma
**Stadlbauer KH 2007**	Hemodynamic variables<br>Survival	Abdominal vascular injury
**Voelckel WG 2003**	Hemodynamic variables<br>Regional blood flow<br>Short-term survival	Liver trauma

Tab. 4: Primary endpoint of animal studies
